# Immunopathogenesis of hidradenitis suppurativa and response to anti–TNF-**α** therapy

**DOI:** 10.1172/jci.insight.139932

**Published:** 2020-10-02

**Authors:** Margaret M. Lowe, Haley B. Naik, Sean Clancy, Mariela Pauli, Kathleen M. Smith, Yingtao Bi, Robert Dunstan, Johann E. Gudjonsson, Maia Paul, Hobart Harris, Esther Kim, Uk Sok Shin, Richard Ahn, Wilson Liao, Scott L. Hansen, Michael D. Rosenblum

**Affiliations:** 1 Department of Dermatology, University of California at San Francisco (UCSF), San Francisco, California, USA.; 2 AbbVie Cambridge Research Center, Cambridge, Massachusetts, USA.; 3 AbbVie Bioresearch Center, Worcester, Massachusetts, USA.; 4 Department of Dermatology, University of Michigan, Ann Arbor, Michigan, USA.; 5 Department of Surgery, UCSF, San Francisco, California, USA.; 6 Institute for Quantitative and Computational Biosciences, University of California at Los Angeles, Los Angeles, California, USA.

**Keywords:** Dermatology, Immunology, Adaptive immunity, Innate immunity, Skin

## Abstract

Hidradenitis suppurativa (HS) is a highly prevalent, morbid inflammatory skin disease with limited treatment options. The major cell types and inflammatory pathways in skin of patients with HS are poorly understood, and which patients will respond to TNF-α blockade is currently unknown. We discovered that clinically and histologically healthy appearing skin (i.e., nonlesional skin) is dysfunctional in patients with HS with a relative loss of immune regulatory pathways. HS skin lesions were characterized by quantitative and qualitative dysfunction of type 2 conventional dendritic cells, relatively reduced regulatory T cells, an influx of memory B cells, and a plasma cell/plasmablast infiltrate predominantly in end-stage fibrotic skin. At the molecular level, there was a relative bias toward the IL-1 pathway and type 1 T cell responses when compared with both healthy skin and psoriatic patient skin. Anti–TNF-α therapy markedly attenuated B cell activation with minimal effect on other inflammatory pathways. Finally, we identified an immune activation signature in skin before anti–TNF-α treatment that correlated with subsequent lack of response to this modality. Our results reveal the fundamental immunopathogenesis of HS and provide a molecular foundation for future studies focused on stratifying patients based on likelihood of clinical response to TNF-α blockade.

## Abstract 2

Hidradenitis suppurativa lesions are characterized by alterations in cDC2s, relatively reduced Tregs, an influx of memory B cells and plasma cells, and biases towards IL1 pathway activation and type 1 T cell responses.

## Introduction

Hidradenitis suppurativa (HS) is a painful and debilitating chronic inflammatory disease with no uniformly effective treatment. It affects 1%–4% of the Western population, a prevalence on par with that of other inflammatory skin diseases, such as psoriasis vulgaris (1, 2). Unlike psoriasis, the immunobiology of HS is poorly understood, which has hindered the development of effective therapies (3). Clinically, HS is characterized by painful inflammatory nodules and abscesses, tunnels with malodorous discharge, and disfiguring scarring involving intertriginous body sites, including the axillae, breasts, groin, and buttocks (4). Although the presence of abscesses and malodorous drainage has implicated microorganisms in HS pathogenesis, antimicrobial therapy has been shown to be inconsistently and often only temporarily effective (5, 6). In recent years, limited studies implicating dysregulated immune responses in HS, including elevations in TNF-α (7–10), IL-1β (9, 11), and IL-12/23 (12, 13), have guided the use of biologic agents for this disease with variable success (14–19). The TNF-α antagonist adalimumab is the first and only US FDA–approved therapy for this disease. While more effective than any of the myriad other agents used for HS management, including broad-spectrum systemic antibiotics, systemic retinoids, and hormonal therapies, adalimumab efficacy for HS remains limited (20). In phase III trials, adalimumab therapy reduced HS lesions by 50% at 12 weeks in less than 45% of study subjects (21). Other available biologic therapies, including anti–IL-1 and anti-IL–12/23 agents, have shown some success in small studies (18, 22, 23). Anti–IL-17A therapeutics for HS are currently under investigation (24, 25).

The majority of ongoing HS clinical trials are focused on “pivoting” therapies developed for other skin diseases (mainly psoriasis) to HS. Although TNF-α blockade is used for management of HS and psoriasis, these 2 inflammatory skin conditions are quite different. Both diseases are notable for keratinocyte hyperproliferation; however, in HS lesions the proliferation invades deeply while the hyperproliferative epithelium of psoriatic lesions tends to remain superficial. In addition, unlike psoriasis, the initial pathogenesis of HS appears to center on hair follicles, leading to deep sinus tracks and dermal fibrosis, features not observed in psoriasis (26, 27). Given the highly inflammatory nature of HS, unique skin pathology, and relative paucity of studies focused on the immunology of this disease, it is clear that foundational knowledge of immune dysregulation in HS is urgently needed to identify specific pathways that may lead to more effective treatments. Identification and functional interrogation of the immune cells and inflammatory mediators driving HS has been limited by the lack of an animal model. Thus, mechanistic studies of HS skin samples, both with and without immunomodulating therapies, remain a critical approach.

Here, we define immune dysfunction in normal appearing skin in HS patients that may predispose to disease. In addition, we uncover the cellular infiltrate and inflammatory pathways that dominate in HS lesions during specific disease stages, before and after anti–TNF-α therapy. In doing so, we provide a molecular rationale for specific combinations of immunomodulatory approaches to treat HS, as well as uncover a molecular signature associated with poor clinical responses to TNF-α blockade.

## Results

### HS skin lesions have a unique inflammatory signature.

**Figure 1:**
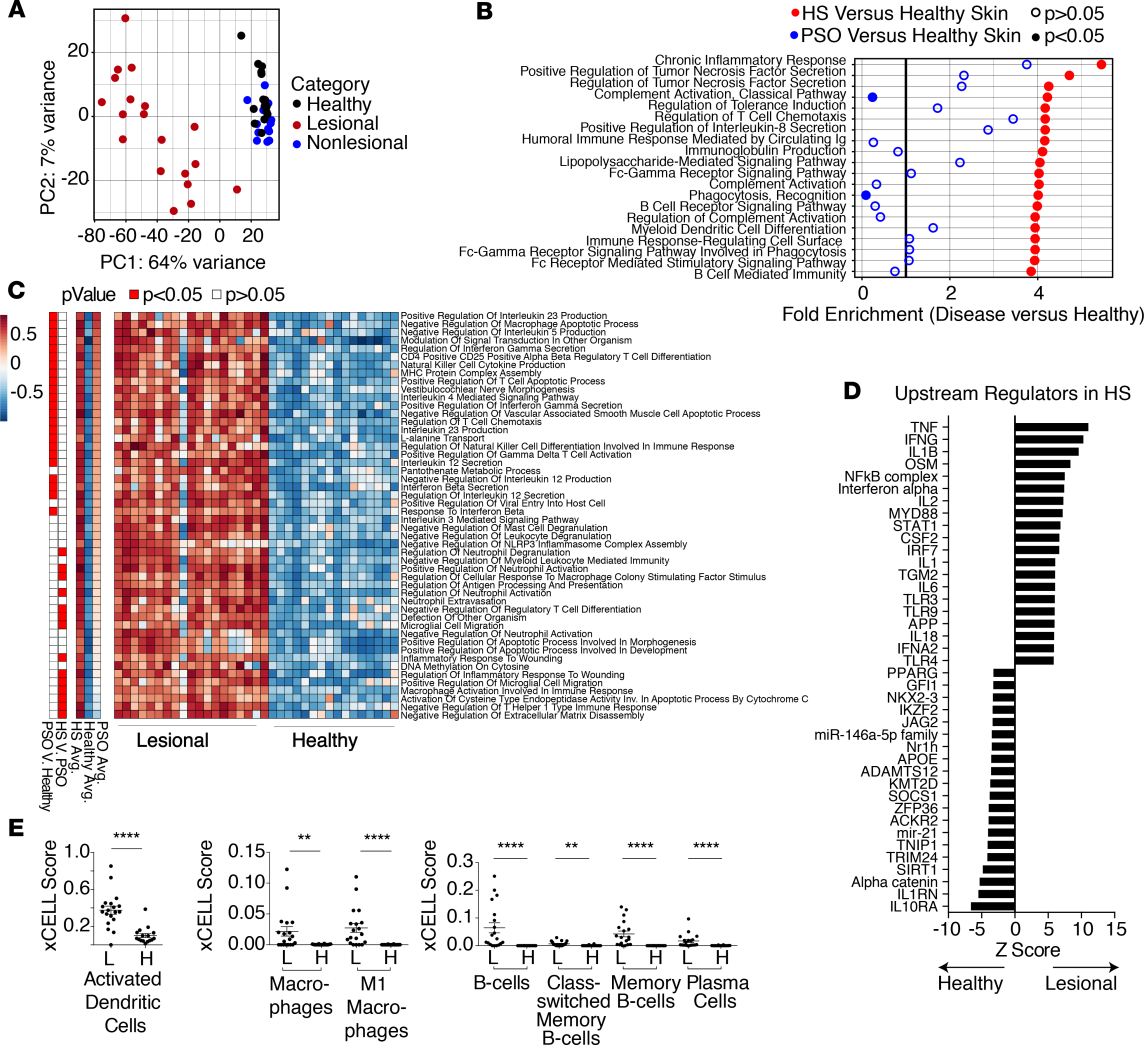
Elucidation of the dominant inflammatory pathways in HS skin. (**A**) Principal components analysis (PCA) of whole-tissue RNA-Seq data from lesional HS skin (*n* = 19), nonlesional HS skin (*n* = 13), and site-matched healthy control skin (*n* = 16). All samples were taken before the initiation of anti–TNF-α therapy. (**B**) The top 20 enriched (FDR < 0.05, Fisher exact with Benjamini-Hochberg correction) PANTHER Gene Ontology pathways identified from genes significantly (adjusted *P* < 0.05, Wald’s test) increased in pretreatment lesional HS skin versus healthy control skin are depicted in red. Fold enrichment of pathways in genes significantly (adjusted *P* < 0.05, Wald’s test) increased in lesional psoriasis skin (*n* = 8) versus healthy control skin (*n* = 9) is depicted in blue. (**C**) Heatmap depicting the Gene Set Variation Analysis (GSVA) enrichment scores of the top 50 significantly different (adjusted *P* < 0.05, empirical Bayes test with Benjamini-Hochberg correction) Gene Ontology pathways in whole-tissue RNA-Seq data of pretreatment HS lesional skin versus healthy control skin. Each column depicts an individual patient. Average pathway enrichment scores in HS skin, normal skin, and psoriatic skin, is depicted (left); pathways significantly different (adjusted *P* < 0.05) comparing HS skin and psoriatic skin are indicated. (**D**) Ingenuity Pathway Analysis (IPA) of upstream regulators significantly (*P* < 0.05) different in lesional HS skin versus healthy controls. (**E**) xCell Scores indicating predicted enrichment of different cell populations in whole-tissue RNA-Seq data from lesional HS (L) and healthy (H) skin. Each dot represents an individual patient. All figure error bars show mean ± SEM. (**P < 0.01, ****P < 0.0001, Mann-Whitney *U* test.)

In order to broadly interrogate immune pathways active in skin of patients with HS, we performed whole-tissue whole-transcriptome RNA-sequencing (RNA-Seq) on 19 lesional and 13 nonlesional skin samples of HS study subjects before initiation of anti–TNF-α therapy. Transcriptional signatures were compared with 16 samples of site-matched healthy control skin and our data set of inflamed skin from patients with conventional psoriasis (28). Principal components analysis (PCA) showed that the transcriptome of lesional HS skin before adalimumab treatment was vastly different from that of nonlesional skin and healthy control skin (Figure 1A). Given the large number of genes significantly altered in HS lesional skin, we interrogated which pathways were broadly perturbed in this tissue relative to healthy control skin. We additionally used our psoriatic lesional skin biopsies and corresponding site-matched healthy control skin as comparators to better discriminate which pathways are specific to HS. PANTHER Gene Ontology analysis revealed the most highly enriched pathways in lesional HS skin to be immune related; however, the breadth of immune compartments involved and the magnitude of inflammatory gene expression were striking and unique to HS (Figure 1B). For example, genes associated with chronic inflammation, TNF-α secretion, recruitment of T cells, and LPS signaling were features of both psoriatic skin disease and HS. However, there was a greater than 4-fold enrichment in the majority of these pathways in HS skin, whereas relatively few reached this degree of enrichment in psoriatic skin (Figure 1B). HS skin uniquely bore signatures of complement activation, B cell signaling, and pathways involving phagocytosis.

To more precisely identify broader changes in inflammatory pathways across the transcriptome and uncover pathways consistently increased in individual patients, we performed Gene Set Variation Analysis (GSVA) on HS lesional skin, psoriatic lesional skin, and healthy control skin (Figure 1C). GSVA score enrichment of gene sets across an individual sample’s transcriptome detects shifts in pathway expression without relying on arbitrary significance cutoffs or collapsing individual variation (29). Using this analysis, we observed that many of the dominant pathways enriched in HS skin were also high in psoriatic lesions, including production of IL-12 and genes involved in T cell chemotaxis. However, multiple pathways, including those involving neutrophil recruitment, macrophage activation, and responses to wounding, were uniquely increased in HS lesional skin (Figure 1C).

We next sought to determine the main drivers of the inflammatory pathways that predominate in HS skin lesions. To do so, we quantified upstream transcriptional regulators using Ingenuity Pathway Analysis (IPA; QIAGEN) to identify the net effect of regulatory molecules within the tissue. When comparing HS lesional skin to healthy control skin, TNF-α–regulated genes were identified as the most highly increased, followed closely by IFN-γ and IL-1β (Figure 1D). Conversely, the IL-1 receptor antagonist, IL-1RN, and IL-10RA, 2 potent immunoregulatory molecules (30, 31), were relatively reduced in HS skin. In addition to immune modulators, α-catenin and sirtuin 1, both important for regulation of cell proliferation and survival, were reduced in HS skin.

To begin to identify the major immune cell types contributing to the HS inflammatory transcriptome, we used the xCELL scoring tool, which predicts cell types present in RNA-Seq data (32). This analysis suggested a predominance of activated dendritic cells and proinflammatory M1 macrophages (Figure 1E). In agreement with our PANTHER pathway analysis (Figure 1B), we observed enhanced B cell signatures. Increased plasma cells, as well as memory B cells, were predicted to significantly contribute to the HS transcriptional signature (Figure 1E), suggesting that these cell types be interrogated further (described below). Taken together, these data suggest that HS skin lesions have a heightened and more heterogenous inflammatory signature compared with psoriasis. In addition, IL-23 and IL-17 were not the highest drivers of the transcriptional changes observed in HS skin.

### Nonlesional skin is abnormal in patients with HS.

**Figure 2:**
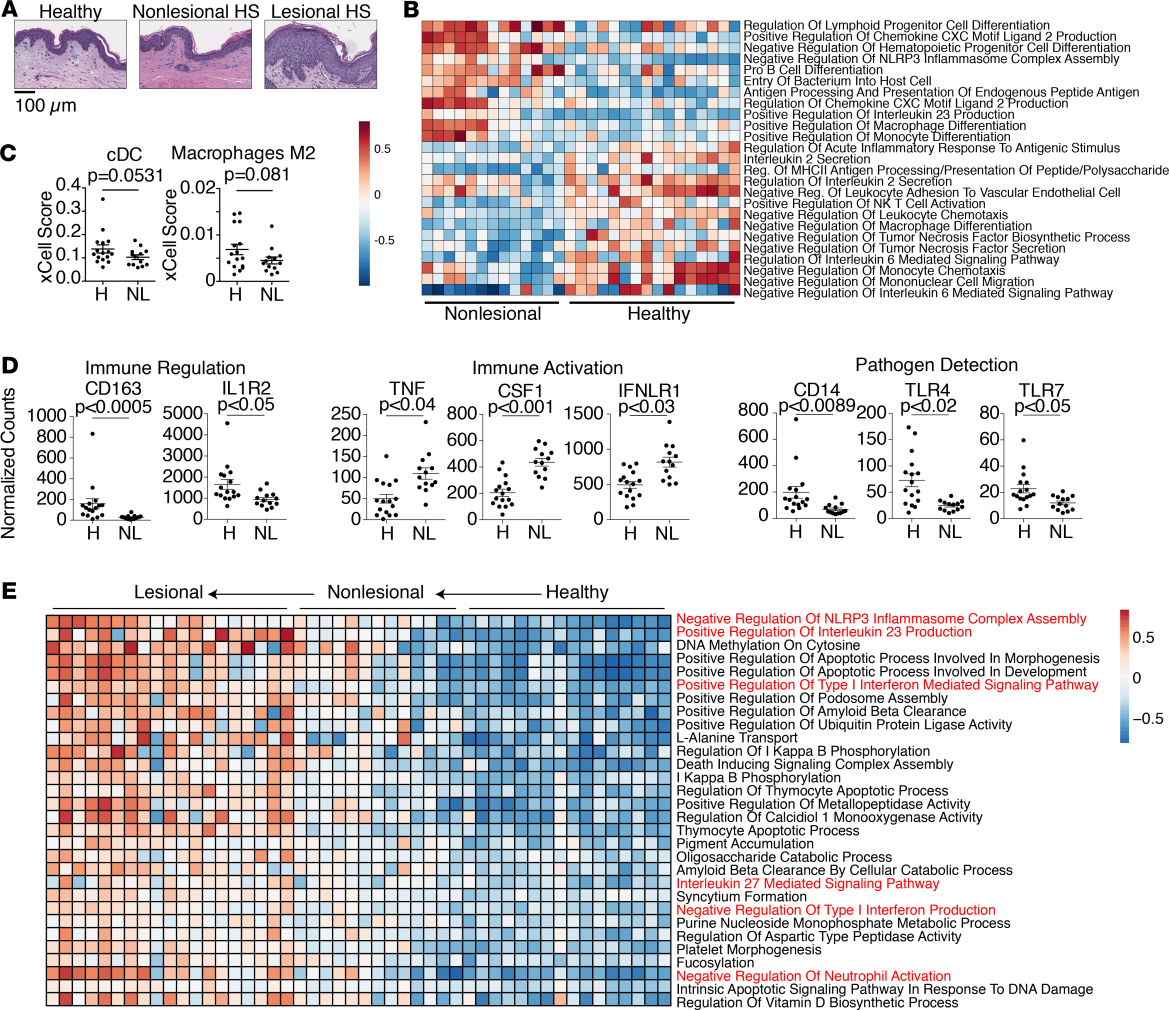
Nonlesional skin in HS has defects in immune regulatory pathways. (**A**) Representative H&E staining of lesional skin, nonlesional skin, and site-matched healthy control skin. (**B**) GSVA enrichment scores of the significantly different (adjusted *P* < 0.05, absolute log fold change > 0.3, empirical Bayes test with Benjamini-Hochberg correction) Gene Ontology immune-related pathways in whole-tissue RNA-Seq data of healthy control skin (*n* = 16) compared with pretreatment nonlesional HS skin (*n* = 13). Each column depicts an individual patient. (**C**) xCell Scores indicating predicted enrichment of different cell populations in whole-tissue RNA-Seq data in nonlesional HS (NL) and healthy (H) skin. Each dot represents an individual patient (Mann-Whitney *U* test) (**D**) Normalized counts for selected transcripts in whole-tissue RNA-Seq comparing healthy control skin with pretreatment nonlesional HS skin (adjusted *P*, Wald’s test). (**E**) GSVA enrichment scores of the union of Gene Ontology pathways significantly increased (adjusted *P* < 0.05, empirical Bayes test with Benjamini-Hochberg correction) in pretreatment lesional HS skin (*n* = 19) versus pretreatment nonlesional HS skin and pretreatment nonlesional HS skin versus healthy control skin. Each column depicts an individual patient.

To determine if normal appearing skin in patients with HS (defined as clinically normal appearing skin more than 10 cm away from an active lesion in the same anatomic location) was abnormal at the molecular level, we compared the transcriptome of the 13 nonlesional skin samples of HS study subjects with age- and site-matched skin from 16 normal healthy controls. Clinically normal appearing nonlesional skin from patients with HS showed no signs of inflammation by routine histology and was indistinguishable from healthy control skin (Figure 2A). Interestingly, nonlesional skin was somewhat distinct from healthy control skin at the transcriptional level by PCA (Supplemental Figure 1; supplemental material available online with this article; https://doi.org/10.1172/jci.insight.139932DS1). Approximately 2300 genes were significantly differentially expressed (adjusted *P* < 0.05) between the 2 tissues. PANTHER pathway analysis showed enriched pathways related to homeostatic processes such as cellular respiration, metabolism, and cell division. However, regulation of IL-1 signaling was an immune process increased in healthy skin relative to nonlesional HS skin (Supplemental Figure 2). GSVA revealed that the majority of immune pathways differentially expressed between healthy skin and HS nonlesional skin involved components of the myeloid compartment. Pathways increased in healthy skin involved negative regulation of monocyte and macrophage activation, while pathways involving CXCL2, a powerful neutrophil chemoattractant (33), were increased in HS nonlesional skin (Figure 2B). Consistent with perturbations observed in nonlesional skin largely involving myeloid cells, xCELL analysis suggested that conventional dendritic cells and alternatively activated M2 macrophages were reduced in HS nonlesional skin (Figure 2C). At the individual transcript level, CD163, a marker of immunoregulatory macrophages (34), was reduced in nonlesional HS skin (Figure 2D). Consistent with IL-1 signaling being a dominant driver of the HS transcriptome (Figure 1D), IL-1 receptor 2 (IL-1R2) was decreased in nonlesional HS skin (Figure 2D). IL-1R2 is well known to be a major negative regulator of IL-1 signaling in tissues (31, 35, 36). In addition, production of specific proinflammatory mediators, such as TNF-α, CSF1, and the type I IFN receptor IFNLR1, was increased in HS nonlesional skin (Figure 2D). Interestingly, nonlesional skin was not simply more “globally activated,” as molecules involved in pathogen detection such as the LPS receptor CD14, as well as TLR4 and TLR7, were reduced in HS nonlesional skin (Figure 2D), as were several antimicrobial peptides (Supplemental Figure 3).

To understand whether inflammatory pathways in HS lesional skin are active in nonlesional HS skin, we compared these transcriptomes with those of healthy control skin (Figure 2E and Supplemental Figure 4). Of the pathways identified to be significant (adjusted *P* < 0.05), many involved regulation of cellular homeostasis, such as apoptosis, metabolism, and DNA damage repair. However, specific inflammatory pathways implicating myeloid cell activation, including those involving the nucleotide-binding domain leucine-rich repeat and pyrin domain containing receptor 3 inflammasome, type I IFN production, neutrophil activation, and monocyte chemokine production, were progressively altered across the spectrum of healthy to nonlesional to lesional HS skin (Figure 2E). Taken together, these results suggest that clinically and histologically normal appearing skin in patients with HS is dysfunctional, with significant reductions in immune regulatory pathways and activation of specific proinflammatory pathways. These data imply that compromised immune regulation and aberrant immune activation may work in concert to reduce the threshold for the initiation of myeloid cell–mediated inflammation in early HS skin lesions.

### Immunoregulatory myeloid cell subsets are reduced and dysfunctional in HS skin.

**Figure 3:**
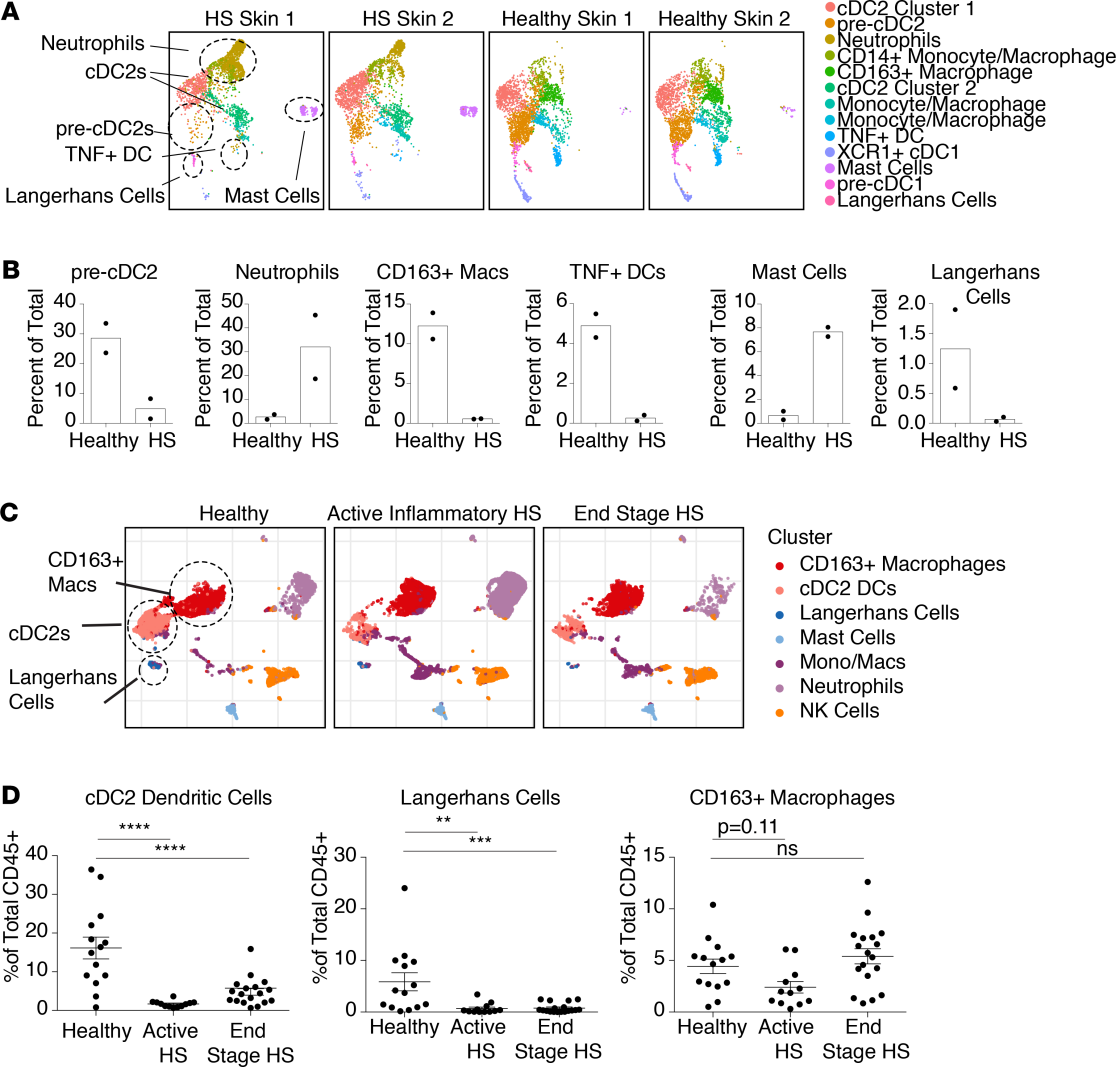
Tissue-infiltrating myeloid cells are dysfunctional in HS skin. (**A**) Uniform manifold approximation and projection (UMAP) plots of scRNA-Seq data of myeloid cells sorted from 2 end-stage HS skin donors versus 2 healthy controls. Plots are equally sampled to 2943 cells per sample. Population identification was manually assigned. (**B**) Percentages of myeloid cell subsets identified in scRNA-Seq data depicted in **A**. (**C**) UMAP plots of myeloid cells immunophenotyped by CyTOF. Cells were pregated on live, singlet, CD45^+^CD3^–^CD19^–^ events and represent 20,442 cells from 7 healthy donors, 38,820 cells from 5 active inflammatory HS lesions, and 8492 cells from 5 end-stage HS surgical resections. (**D**) Percentages of myeloid subsets identified via manual gating in 14 healthy donors, 12 biopsies from active HS lesions, and 18 end-stage HS surgically resected specimens. (*P < 0.05, **P < 0.01, ****P < 0.001, 1-way ANOVA.)

Our initial bulk cell RNA-Seq discovery approach suggested that myeloid cells are major contributors to the overall inflammatory response observed in HS skin. To better understand the myeloid compartment in these lesions at both the transcriptional and protein levels, we performed single-cell RNA-Seq (scRNA-Seq) and mass cytometry by time of flight (CyTOF). Myeloid cells (defined as live CD45^+^CD3^–^CD19^–^ cells) were sort purified for scRNA-Seq from lesional skin of HS patients and healthy controls (Figure 3A and Supplemental Figure 5). As expected, our single-cell data revealed neutrophils to be relatively enriched in HS skin (Figure 3, A and B). The predominant myeloid cell population in normal skin was type 2 conventional dendritic cells (cDC2s), as previously reported (37). While the relative proportion of cDC2s was not dramatically altered between HS and normal skin, a neighboring cluster transcriptionally resembling cells of a pre-cDC2 phenotype (38) was greatly reduced in HS skin (Figure 3, A and B). cDC2s have been implicated in multiple immune functions, including acting synergistically with Tregs to maintain T cell tolerance in tissues (39–42). Interestingly, pre-cDC2 cells expressed higher levels of known immunoregulatory proteins, including indoleamine 2,3-dioxygenase 1, programmed cell death ligand 1, and heme oxygenase 1, when compared with the 2 clusters resembling more classical cDC2s (Supplemental Figure 6). Additionally, expression of a defining marker for cDC2s, CD1c, was diminished in HS skin, further indicating the perturbation of these cells in this tissue (Supplemental Figure 7). Other myeloid clusters, including immunoregulatory macrophages expressing the marker CD163, were proportionally reduced in HS skin. Interestingly, a putative dendritic cell population whose major distinguishing feature was TNF-α expression was diminished in HS skin. The functional role of this myeloid cell subset has yet to be elucidated.

To further define myeloid cells in HS skin at the protein level over a broader range of patients during different stages of disease, we performed 37-parameter CyTOF (Supplemental Figure 8). HS skin lesions stereotypically progress from an acute inflammatory phase to a chronic inflammatory phase to end-stage fibrotic disease (43). Thus, skin was harvested from 12 active inflammatory HS lesions, 18 end-stage fibrotic HS lesions, and 14 site-matched healthy controls. Unbiased clustering revealed similar myeloid cell subsets to those observed in our scRNA-Seq data (Figure 3C). Interestingly, total cDC2 cells, which most likely include the pre-cDC2 population observed in scRNA-Seq data, and Langerhans cells, were significantly reduced in HS skin before end-stage disease (Figure 3D). In addition, CD163^+^ macrophages trended down in active inflammatory lesions and rebounded in end-stage disease to levels observed in healthy skin (Figure 3D).

**Figure 4:**
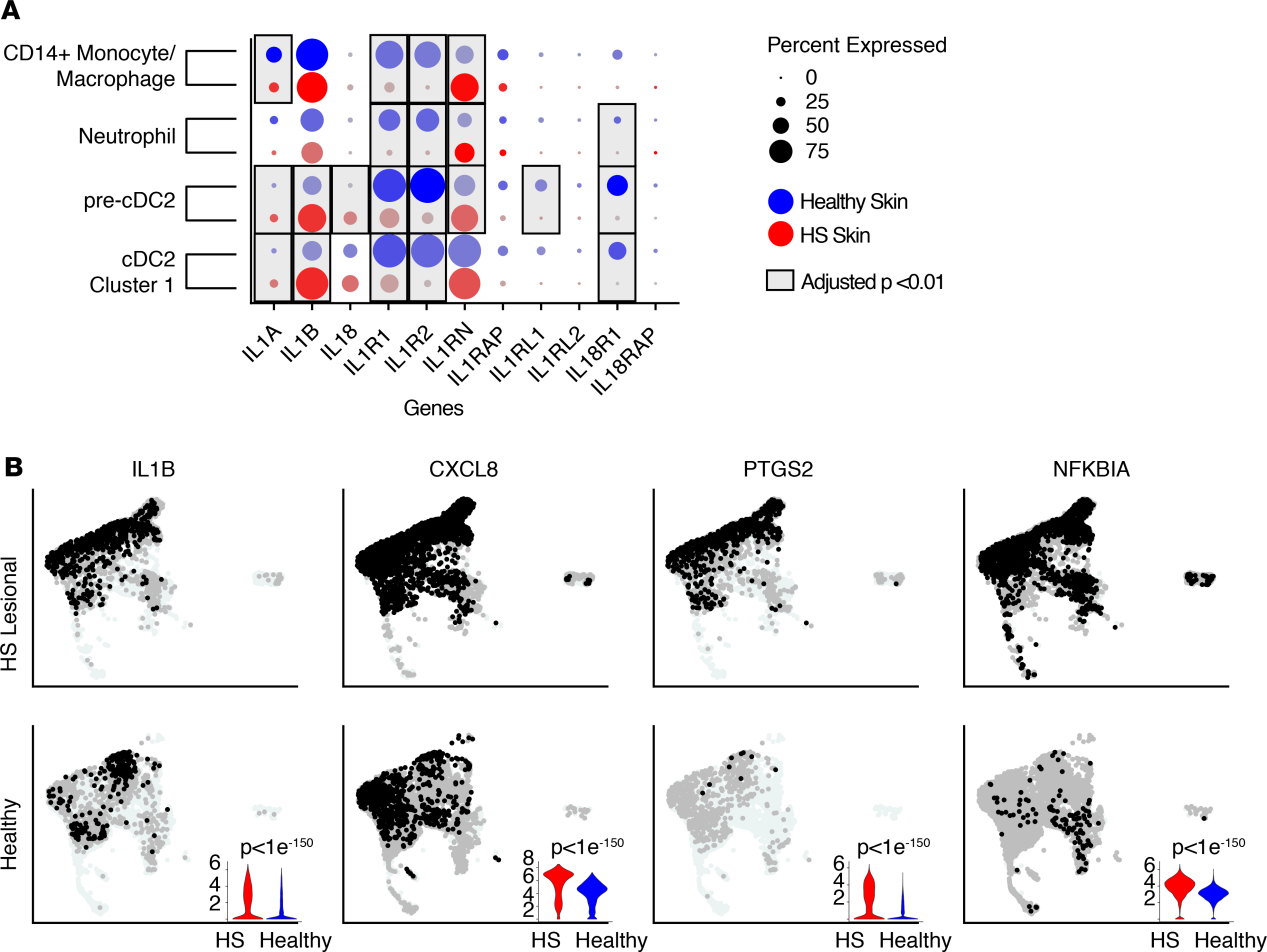
IL-1 signaling is increased in myeloid cells of HS skin. (**A**) Dot plot depicting expression of IL-1 family genes in scRNA-Seq of the top 4 most abundant myeloid clusters. Data averaged from 2 HS skin samples are depicted in red and from 2 healthy skin samples in blue. Dot size indicates percentage expression within clusters, while color intensity indicates degree of expression. Boxed dots indicated an adjusted *P* < 0.01 for the comparison between healthy and HS skin (Wilcoxon’s rank sum test). (**B**) UMAP plots showing intensity of expression of IL-1B and 3 genes downstream of IL-1 signaling (CXCL8, PTGS2, and NFKBIA). Data are combined for 2 HS donors and for 2 healthy controls, and plots are equally sampled to 5886 cells per donor type. Violin plots indicating expression level over the entire population are inset in the lower right for each gene (Wilcoxon’s rank sum test).

These results suggest that there are major quantitative changes in regulatory myeloid cell subsets in HS skin. To determine if these cell subsets are functionally altered, we quantified transcriptional changes in the major myeloid cell populations identified. Multiple immune pathways, including neutrophil chemotaxis, IFN-γ production, and antigen receptor signaling, were increased in HS myeloid cells (Supplemental Figure 9). However, we focused on the IL-1 pathway, given its strong association with myeloid cell biology and the fact that this pathway was fundamentally altered in HS skin at the tissue level (Figure 1 and Figure 2). IL-1β was expressed at similar levels in monocyte/macrophages and neutrophils between HS and healthy skin. However, there was a marked increase in expression of this cytokine in both cDC2s and pre-cDC2s in HS lesions (Figure 4A). In addition, expression of the IL-1 signaling antagonist, IL-1R2, was significantly reduced in the major myeloid subsets in HS skin. IL-1R1, a major component of the IL-1 receptor, was also significantly reduced in these myeloid subsets in HS skin (Figure 4A). However, decreased expression of this receptor appeared to play little role in attenuating IL-1 signaling in these cells, as genes known to be induced upon engagement of this pathway (CXCL8, prostaglandin-endoperoxide synthase 2 [PTGS2], and NF-κB inhibitor alpha [NFKBIA]) (44) were significantly increased in these and other myeloid cells in HS skin (Figure 4B). Taken together, our results suggest that specific regulatory myeloid cell populations are quantitatively reduced in HS skin. Furthermore, the cells within these subsets that remain in skin lesions seem to adopt a proinflammatory phenotype with marked activation of the IL-1 pathway.

### The B cell compartment increases as HS skin lesions evolve.

**Figure 5:**
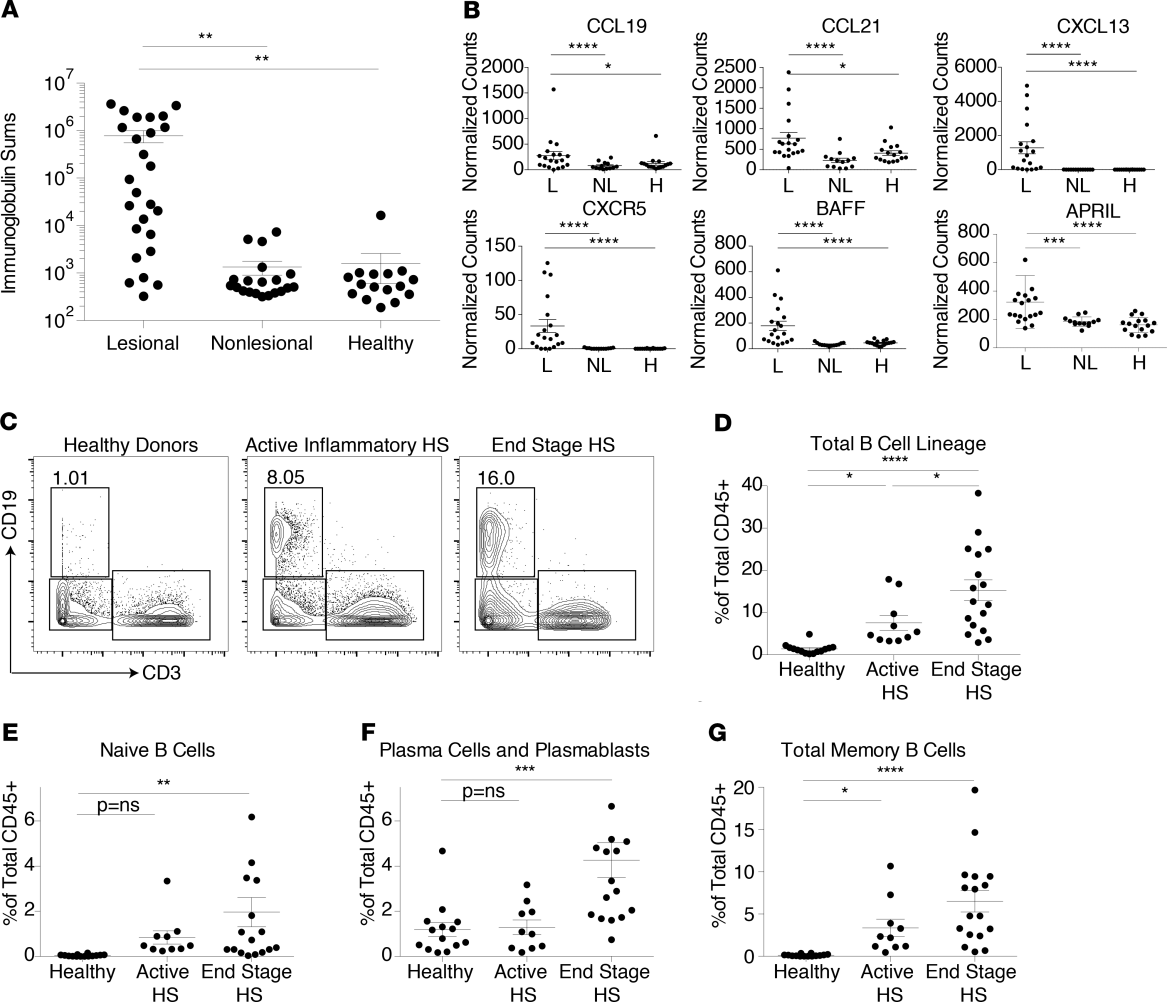
HS progression is associated with a transition from skin-infiltrating memory B cells to plasma cells. (**A**) Total normalized counts of immunoglobulin genes in whole-tissue RNA-Seq data from lesional (L, *n* = 19) and nonlesional (NL, *n* = 13) HS patients before anti–TNF-α therapy and healthy (H, *n* = 16) controls. (**P < 0.01, 1-way ANOVA.) (**B**) Normalized counts for selected B cell chemokine, chemokine receptor, and B cell survival factors in whole-tissue RNA-Seq before anti–TNF-α therapy (*P < 0.05; ***P < 0.005; ****P < 0.001, Wald’s test, DESeq). (**C**) Representative CyTOF plot of CD19 versus CD3 expression in cells from healthy donor skin, biopsied active inflammatory HS lesions, or end-stage HS skin (*n* = 17). Cells are pregated on live, singlet, CD45^+^ events. (**D**–**G**) Percentages of total B cells (**D**), naive B cells (**E**), plasma cells and plasmablasts (**F**), and memory B cells (**G**) among total live, singlet, CD45^+^ events in CyTOF data sampled from healthy donors (*n* = 14), active HS lesion biopsies (*n* = 10), and end-stage HS surgical resections (*n* = 18). (*P < 0.05, **P < 0.01, ***P < 0.005, ****P < 0.001, 1-way ANOVA.)

In addition to myeloid cells, our initial whole-tissue discovery approach suggested that B cells are a major contributor to the HS transcriptional signature (Figure 1). Thus, we further explored the B cell compartment in HS skin via bulk cell RNA-Seq and 37-parameter CyTOF (Supplemental Figure 10). Total immunoglobulin transcripts were significantly increased in lesional HS skin before initiation of anti–TNF-α therapy relative to both nonlesional HS skin and healthy control skin (Figure 5A). In probing our RNA-Seq data set for factors important for B cell recruitment and survival during inflammation, we observed a pronounced increase in CXCL13 expression and its corresponding receptor CXCR5 in HS lesional skin (Figure 5B). CXCL13 has been reported as a dominant driver of B cell infiltration into tissues in other inflammatory settings (45). Conversely, these transcripts were nearly undetectable in nonlesional and healthy skin. The B cell survival factor BAFF and a proliferation-inducing ligand (APRIL) were also increased in HS lesional skin, indicating that inflamed HS skin provides a niche for the persistence of these cells (Figure 5B). Other B cell chemokines, such as CCL19 and CCL21, were less dramatically increased, and CXCL12 was not differentially expressed between HS and healthy skin (Supplemental Figure 11). To determine if myeloid cell subsets were capable of supporting B cells in skin lesions, we probed our myeloid cell scRNA-Seq data and observed that production of the ligand BAFF was elevated in some subsets of myeloid cells in HS skin (Supplemental Figure 12).

Profiling of HS skin via CyTOF confirmed a striking increase of B cells within HS lesions (Figure 5C). The frequency of total B cells within the immune compartment was significantly increased in both active inflammatory lesions taken before anti–TNF-α treatment and end-stage disease, with a significant increase in this cell lineage in end-stage fibrotic tissue compared with acutely inflamed lesional skin (Figure 5, C and D). Our multiparameter approach enabled a relatively detailed classification of the B cell lineage. A significant increase of naive B cells was observed in end-stage disease, with these cells only trending upward in active inflammatory lesions (Figure 5E). Plasma cells and plasmablasts were also most dramatically increased in end-stage disease, with active inflammatory lesions showing little to no increase relative to healthy control skin (Figure 5F). However, memory B cells were significantly increased in active inflammatory disease and continued to be elevated in end-stage HS (Figure 5G). Taken together, these results suggest that B cells progressively increase in relative proportion of the immune cell infiltrate as HS skin lesions evolve, with further differentiated memory B cells, plasma cells, and plasmablasts preferentially associated with end-stage fibrotic lesions.

### T cells are type 1 polarized and Tregs are relatively reduced in HS skin.

**Figure 6:**
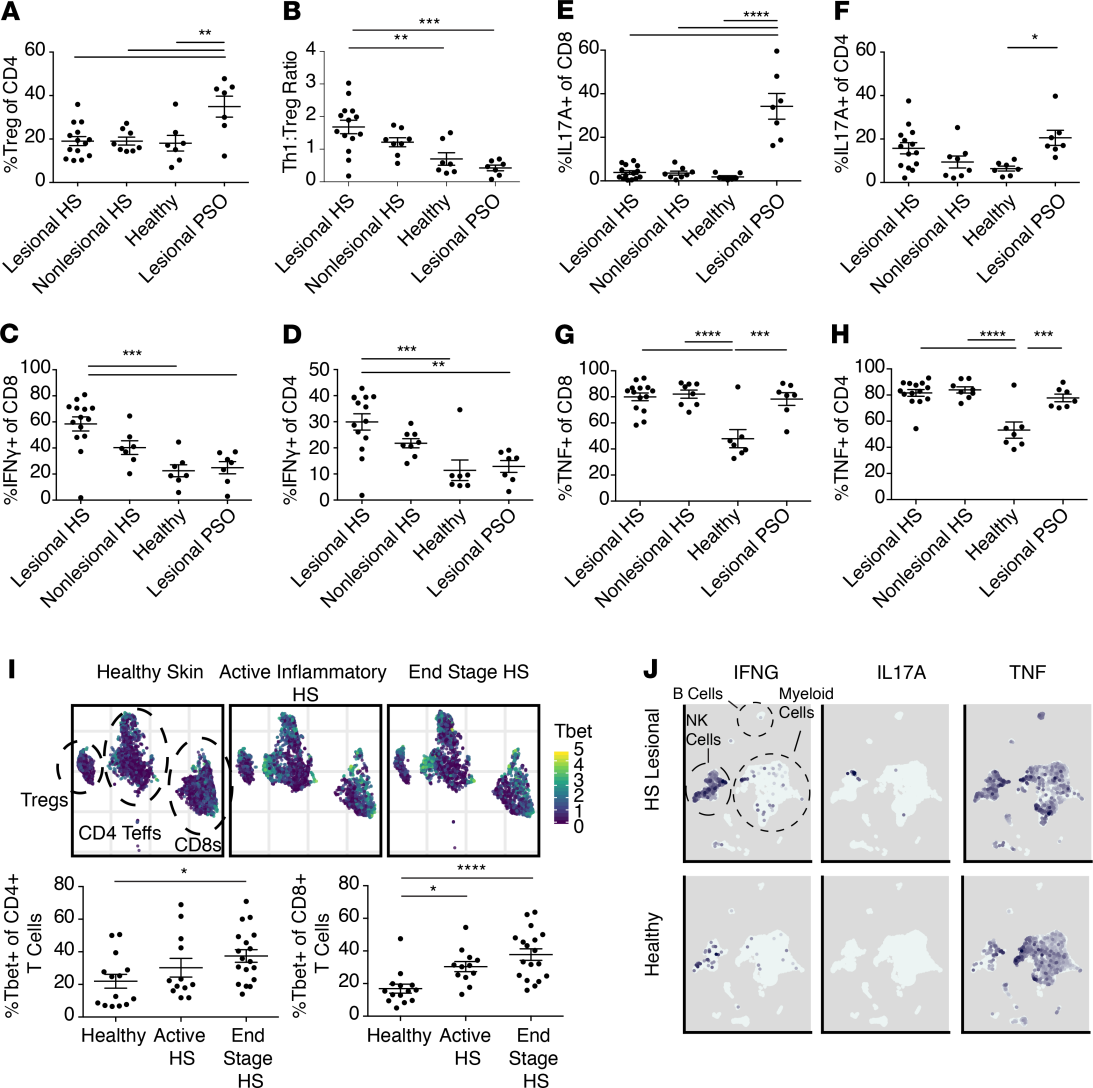
Type 1 T cell responses are dominant in HS skin. (**A**) Flow cytometric quantification of Treg percentages within the CD4^+^ T cell compartment within lesional (*n* = 14) and nonlesional skin (*n* = 8) of patients with HS before anti–TNF-α treatment compared with healthy controls (*n* = 7) and lesional skin from patients with psoriasis (*n* = 7). (**B**) Flow cytometric quantification of ratios of CD4^+^ Th1 cells to Tregs of patients described in **A**. (**C**) Flow cytometric quantification of IFN-γ production within the CD8^+^ T cell compartment of patients described in **A**. (**D**) Flow cytometric quantification of IFN-γ production within the CD4^+^ Tcon compartment of patients described in **A**. (**E**) Flow cytometric quantification of IL-17A production within the CD8^+^ T cell compartment of patients described in **A**. (**F**) Flow cytometric quantification of IL-17A production within the CD4^+^ Tcon compartment of patients described in **A**. (**G**) Flow cytometric quantification of TNF-α production within the CD8^+^ T cell compartment of patients described in **A**. (**H**) Flow cytometric analysis of TNF-α production within the CD4^+^ Tcon compartment of patients described in **A**. (**I**) UMAP plots of CD3^+^ T cells immunophenotyped by CyTOF illustrating intensity of T-bet expression. Cells were pregated on live, singlet, CD45^+^CD3^+^ events and represent 34,432 cells from 7 healthy donors, 75,400 cells from 5 active inflammatory HS lesions, and 21,557 cells from 5 end-stage HS surgical resections. Percentage of T-bet^+^ events within CD4^+^ and CD8^+^ T cells. Data were manually gated for 14 healthy donors, 12 active inflammatory HS lesions, and 18 end-stage surgical resections. (**J**) UMAP plots of scRNA-Seq data of all cells obtained from a myeloid-enriching sort from 2 end-stage HS skin donors versus 2 healthy controls. Intensity of expression of IFN-γ, IL-17A, and TNF-α in all populations is depicted in each plot. (*P < 0.05, **P < 0.01, ***P < 0.005, ****P < 0.001, 1-way ANOVA.)

Our group and others have observed T cells to be the dominant immune cells infiltrating psoriatic plaques (46–48). Given that some similarities exist between inflamed psoriasis and HS skin (Figure 1) and several immunomodulatory therapies approved for the treatment of psoriasis are currently being tested in HS, we functionally defined the T cell compartment in HS skin relative to inflamed psoriasis skin. Using multiparameter flow cytometry, we found no differences in the CD4/CD8 ratio in HS lesional and nonlesional skin compared with both healthy control skin and inflamed (i.e., lesional) psoriatic skin collected contemporaneously (Supplemental Figure 13) (28). Regulatory T cells (Tregs) are a major regulatory cell population in human skin, and our group, as well as others, have observed these cells to be increased in psoriatic lesions (49, 50). In fact, Tregs have been observed to be increased in most inflamed tissues examined in mice and humans (51). This is thought to be due to a compensatory regulatory response because several inflammatory mediators can recruit and activate these cells (52, 53). Interestingly, Tregs were not increased in highly inflamed lesional HS skin (showing similar percentages to that observed in nonlesional HS skin and healthy control skin) and were significantly reduced relative to psoriatic skin (Figure 6A). This lack of Treg expansion resulted in a relative imbalance between these cells and IFN-γ–producing conventional CD4^+^ T cells (i.e., Th1 cells), with the Th1/Treg ratio increased approximately 3-fold in HS skin compared with psoriatic skin (Figure 6B). Intracellular cytokine staining revealed heightened production of IFN-γ in both the CD8^+^ T and conventional CD4^+^ (Tcon) compartments, which was markedly elevated over that observed in psoriasis skin (Figure 6, C and D). Nonlesional HS skin trended toward increased percentages of IFN-γ–producing T cells, although this did not reach statistical significance. Consistent with recent reports (46, 54), IL-17–producing CD8^+^ T cells (i.e., Tc17 cells) comprised a major subset of CD8^+^ T cells infiltrating psoriatic plaques, with approximately 40% of CD8s producing IL-17A compared with approximately 20% of CD4^+^ Tcons producing this cytokine in these skin lesions (Figure 6, E and F). Interestingly, CD8^+^ T cells infiltrating HS skin did not make appreciable amounts of IL-17A, whereas CD4^+^ Tcons in lesional HS skin produced similar levels of this cytokine to that of psoriasis (Figure 6, E and F). In both lesional HS and psoriatic skin, approximately 80% of CD8^+^ T cells and CD4^+^ Tcons produced TNF-α, compared with approximately 50% observed in healthy control skin (Figure 6, G and H). Interestingly, both CD8^+^ T cells and CD4^+^ Tcons in nonlesional HS skin produced TNF-α to the same extent as that observed in inflamed lesional HS and psoriatic skin (Figure 6, G and H). Thus, the propensity of T cells to produce elevated levels of TNF-α may serve as another indication that immune activation is occurring in nonlesional HS skin before clinical and histologic evidence of overt inflammation.

To determine if type 1 T cell polarization correlated with disease stage, we used CyTOF to quantify levels of the master type 1 transcription factor, T-bet, in T cells isolated from healthy control skin, inflamed lesional HS skin, and end-stage HS skin. This analysis revealed increased T-bet levels in inflamed HS skin that trended higher with disease progression (Figure 6I). Using our myeloid scRNA-Seq data set, we asked whether IL-17A, IFN-γ, or TNF-α was produced by other immune cell populations within the HS skin (Figure 6J). This analysis revealed increased IFN-γ expression in a cluster of putative NK cells and TNF-α expression in NK cells, B cells, and myeloid cells (Figure 6J). IL-17A was not detected at appreciable levels in any of these immune cell subsets. Taken together, these data suggest that the T cell compartment in HS skin is type 1 polarized, with a relative reduction in Tregs when compared with psoriatic skin. In addition, T cells are capable of producing increased amounts of IFN-γ and TNF-α in nonlesional HS skin.

### Anti–TNF-α therapy preferentially attenuates the B cell infiltrate in HS skin.

**Figure 7:**
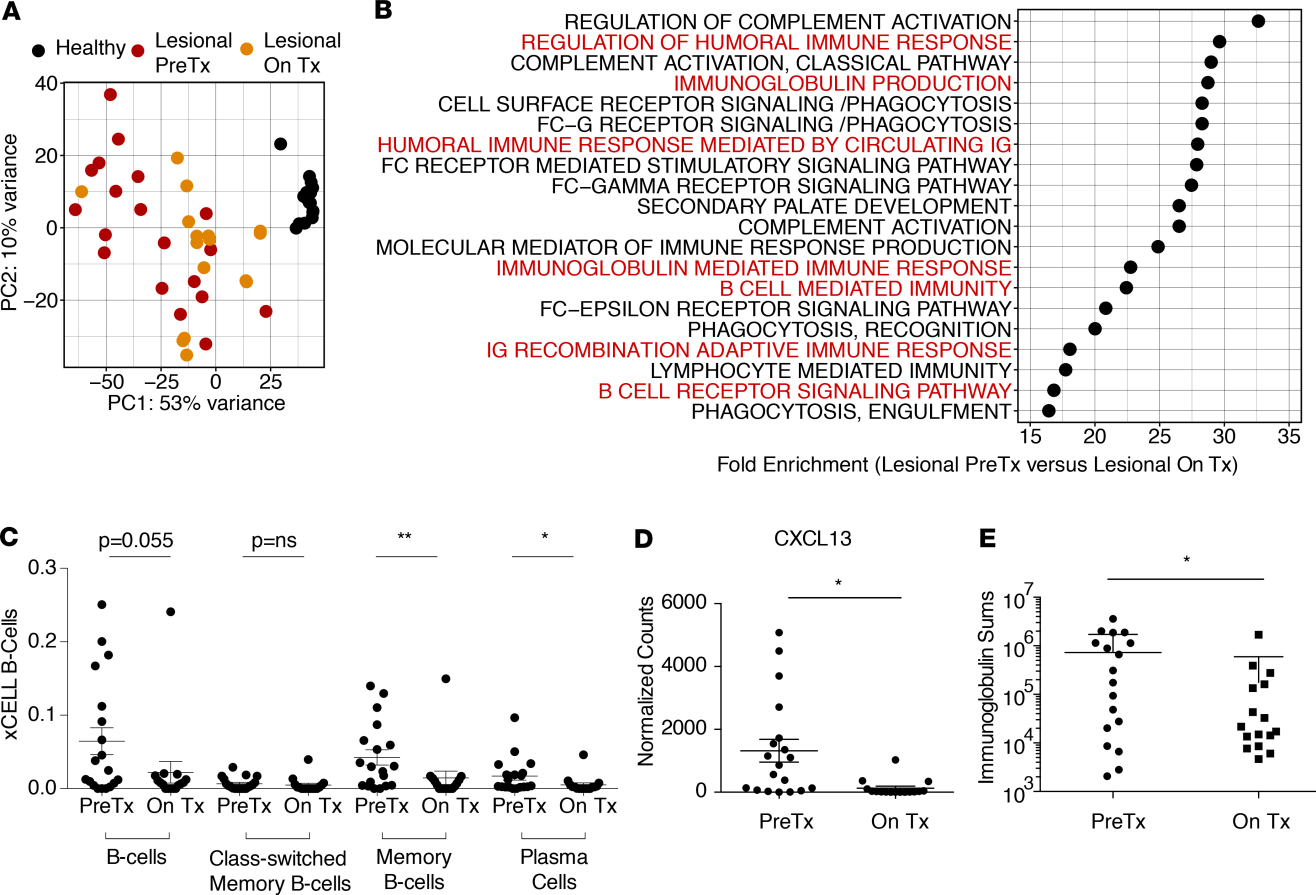
Anti–TNF-α therapy preferentially attenuates the B cell response. (**A**) PCA of RNA-Seq data from lesional skin taken from HS patients before initiation of anti–TNF-α therapy (PreTx, *n* = 19) and on anti–TNF-α therapy (On Tx, *n* = 16), as well as site-matched healthy controls (*n* = 16). (**B**) The top 20 enriched (FDR < 0.05, Fisher exact with Benjamini-Hochberg correction) PANTHER Gene Ontology Pathways identified from genes significantly (adjusted *P* < 0.05, Wald’s test) increased in pre–anti–TNF-α lesional HS skin compared with lesional skin of patients on anti–TNF-α treatment. B cell–related pathways are highlighted in red. (**C**) xCell scores comparing predicted enrichment of B cell subsets in whole-tissue RNA-Seq data of pretreatment patients compared with patients on anti–TNF-α therapy. Each dot represents an individual patient. (*P < 0.05, **P < 0.01, Mann-Whitney *U* test.) (**D**) Normalized counts of CXCL13 transcripts in whole-tissue RNA-Seq of lesional skin before anti–TNF-α therapy versus on anti–TNF-α therapy (*P < 0.05, Wald’s test). (**E**) Total counts of immunoglobulin genes in whole-tissue RNA-Seq from lesional HS patients before anti–TNF-α therapy versus patients on treatment (*P < 0.05, unpaired *t* test).

**Figure 8:**
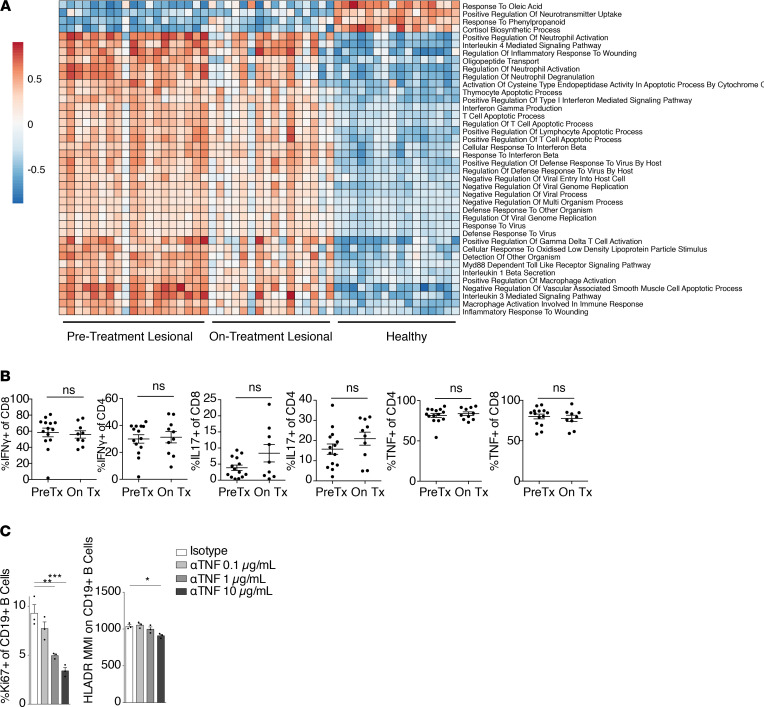
Anti–TNF-α therapy has minimal effects on IL-1 and type 1 T cell inflammation. (**A**) Heatmap depicting the GSVA enrichment scores of top 40 Gene Ontology pathways in whole-tissue RNA-Seq data that were significantly (adjusted *P* < 0.05, empirical Bayes test with Benjamini-Hochberg correction) increased or decreased when comparing pretreatment HS lesional skin (*n* = 19) with healthy controls (*n* = 16) and were also significantly (adjusted *P* < 0.05) increased or decreased in comparison of on-treatment HS lesional skin (*n* = 16) with healthy controls. Each column depicts an individual patient. (**B**) Flow cytometric analysis of indicated cytokines within the CD4^+^ Tcon or CD8^+^ compartments comparing lesional skin of patients before anti–TNF-α treatment (PreTx, *n* = 14) versus patients on anti–TNF-α treatment (On Tx, *n* = 9). (ns, nonsignificant, unpaired *t* test.) (**C**) Percentage of Ki67^+^ of CD19^+^ B cells and HLA-DR median metal intensity following 3 days of culture with either isotype control antibody or increasing doses of anti–TNF-α antibody. Each dot represents a culture well for a single donor sample. Ki67 data are representative of 3 separate donors. (*P < 0.05, **P < 0.01, ***P < 0.005, 1-way ANOVA.)

Given the magnitude and heterogeneity of the inflammatory response in skin of HS patients (Figure 1), we sought to determine how anti–TNF-α therapy altered the immune milieu in this skin. To do so, we performed bulk cell RNA-Seq and multiparameter flow cytometry on patients with HS before and after the initiation of adalimumab. PCA of the whole-skin transcriptome of 19 HS lesions before initiation of therapy and 16 on adalimumab revealed that this intervention shifted the transcriptome to more closely resemble that of site-matched normal healthy control skin; however, a relatively large amount of variability was observed (Figure 7A). PANTHER analysis comparing pretreatment and on-treatment skin revealed a preferential reduction in B cell pathway genes (Figure 7B). A decrease in complement activation and phagocytosis pathways was also observed after treatment (Figure 7B). Consistent with these findings, xCELL analysis of pretreatment and on-treatment skin suggested that anti–TNF-α therapy induced a marked reduction in the B cell compartment, with the largest effect observed in memory B cells and plasma cells (Figure 7C). CXCL13 was significantly decreased in patients on therapy, and BAFF trended down after treatment (Figure 7D and Supplemental Figure 14). In addition, the total immunoglobulin transcript count was significantly reduced in treated patients (Figure 7E). Interestingly, anti–TNF-α therapy resulted in a relative preferential attenuation of B cell–specific gene expression. GSVA revealed that the overwhelming majority of other inflammatory pathways were unchanged after this treatment, including neutrophil activation, type I IFN production, IFN-γ production, and IL-1β secretion (Figure 8A). Consistent with these results, flow cytometric quantification of intracellular cytokine expression in T cells isolated from HS skin revealed no differences in IFN-γ, TNF-α, and IL-17A production in the CD8^+^ and CD4^+^ compartments after initiation of anti–TNF-α therapy (Figure 8B).

To functionally validate that anti–TNF-α therapy attenuates B cell activation in HS skin, we employed a potentially novel ex vivo HS skin culture approach (Supplemental Figure 15). We cultured single-cell suspensions obtained from enzymatically digested HS skin for 3 days in the presence of low concentrations of anti-CD3 and anti-CD28 antibodies. Inflamed skin from patients with HS was cultured with increasing concentrations of adalimumab or isotype control antibody for 3 days and B cell activation quantified by CyTOF. Consistent with our results on patients with HS treated with adalimumab, this treatment induced a dose-dependent decrease in B cell activation, as measured by a reduction in proliferation marker Ki67 and the activation marker HLA-DR (Figure 8C) across multiple donors (Supplemental Figure 16). Taken together, these results suggest that anti–TNF-α treatment preferentially attenuates the B cell component of the inflammatory response in HS skin.

### Lack of clinical response to anti–TNF-α therapy correlates with heightened immune cell recruitment and B cell molecular signatures.

**Figure 9:**
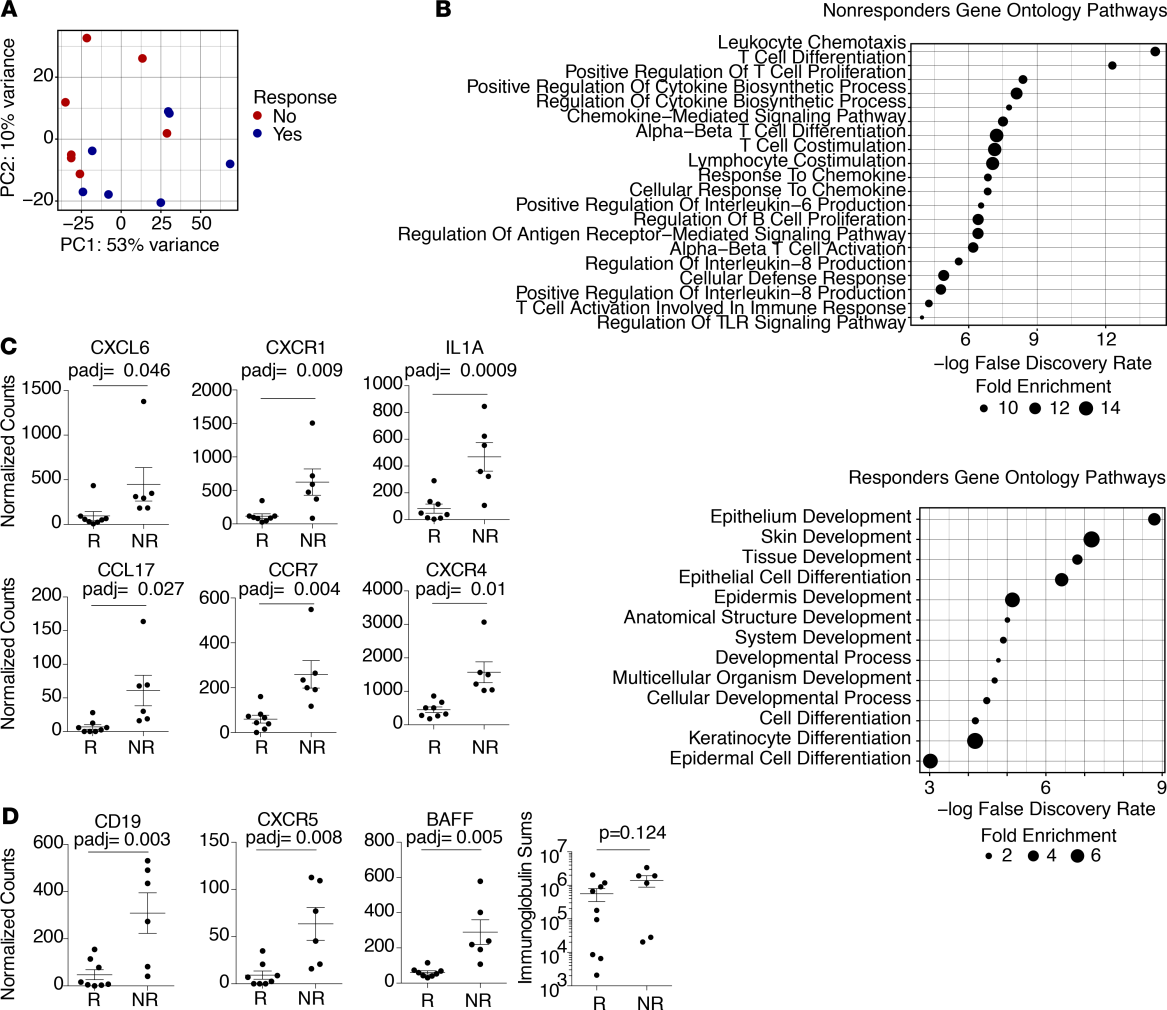
Response to anti–TNF-α therapy correlates with lower B cell and leukocyte chemotaxis immune signatures. (**A**) PCA of RNA-Seq data from lesional skin of patients with HS before initiation of anti–TNF-α therapy comparing patients who later responded to therapy (*n* = 7) and those who did not (*n* = 7). (**B**) The top 20 enriched (FDR < 0.05, Fisher exact with Benjamini-Hochberg correction) PANTHER Gene Ontology pathways identified from genes significantly (adjusted *P* < 0.05, Wald’s test) increased (top) or decreased (bottom) in lesional HS skin of nonresponders (NR) versus responders (R) to anti–TNF-α therapy. (**C**) Normalized counts for selected transcripts in whole-tissue RNA-Seq comparing responders to anti–TNF-α therapy with nonresponders (Wald’s test). (**D**) Normalized counts for selected B cell–related transcripts (left, Wald’s test) and total counts of immunoglobulin genes (right, unpaired *t* test) and comparing responders to anti–TNF-α therapy with nonresponders.

Clinical response to anti–TNF-α therapy in HS is suboptimal, with approximately half of treated patients failing to respond to the FDA-approved dose of adalimumab (21). The ability to predict which patients have an increased chance of benefiting from this treatment, as well as those with lower potential for response, would help stratify clinical risk/benefit for individual patients. In an attempt to elucidate whether specific transcriptional signatures in HS skin correlate with response to TNF-α blockade, we compared whole-transcriptome gene expression in our pretreatment bulk RNA-Seq data set with subsequent response to adalimumab therapy. Partial responders and nonresponders were combined into a single group for this analysis because objective clinical disease of partial responders was not significantly reduced by adalimumab based on achievement of Hidradenitis Suppurativa Clinical Response (HiSCR) (55). Importantly, pretreatment lesional HS skin specimens were clinically indistinguishable between patients who eventually went on to respond or not respond to this treatment. PCA revealed that nonresponders largely clustered apart from responders (Figure 9A). Approximately 420 genes were significantly differentially expressed in pretreatment samples between patients who subsequently responded or did not respond to the FDA-approved dose of adalimumab (Supplemental Figure 17). PANTHER pathway analysis showed genes elevated in nonresponders were largely immune related, involving leukocyte chemotaxis, T cell cytokine production regulation of B cell proliferation, and IL-6/IL-8 expression (Figure 9B). In contrast, genes significantly increased in responders involved pathways pertaining to epithelial and skin development as well as keratinocyte differentiation (Figure 9B). Thus, despite both being highly inflamed skin at the time of pretreatment analysis, patients who subsequently responded to anti–TNF-α therapy had a reduced inflammatory signature and a heightened skin-regenerative signature when compared with pretreatment skin specimens from patients who subsequently did not respond or only partially responded to this treatment. The neutrophil chemokine CXCL6 and its receptor CXCR1 (56) were both elevated in nonresponders, as was IL-1α, while IL-1β expression trended higher in nonresponders (Figure 9C and Supplemental Figure 18). In addition, several chemoattractants and receptors controlling myeloid cell migration in inflammation, CCL17, CCR7, and CXCR4, were significantly increased in nonresponders (Figure 9C) (57).

Given that anti–TNF-α treatment preferentially attenuates the B cell compartment in HS skin (Figure 7 and Figure 8), we asked whether B cell–specific genes were differentially expressed in pretreatment skin between patients who subsequently responded or did not respond to this therapy. Interestingly, we observed a trend toward a greater presence of immunoglobulin transcripts in nonresponders, along with significantly increased expression of CD19, CXCR5, and BAFF (Figure 9D). Thus, patients with evidence of a heightened B cell infiltrate in skin before anti–TNF-α therapy may be less likely to respond. Taken together, these data suggest that HS skin lesions with a heightened inflammatory molecular signature, specifically pertaining to immune cell recruitment and B cell infiltration, are less likely to clinically respond to adalimumab. These results provide the foundation for future prospective clinical studies whereby patients can be stratified pretreatment based on a combination of genes elucidated in our data set, in an attempt to validate whether the magnitude of this signature can predict response to TNF-α blockade.

## Discussion

The pathophysiology of HS is complex and not fully understood. It is believed that skin lesions begin with hair follicle occlusion leading to the formation of intradermal follicular cysts that eventually rupture. Ruptured cysts release hair keratins and most likely skin microbes into the dermis, inciting a potent inflammatory response. This process occurs relatively frequently in healthy individuals (termed furuncles in the medical vernacular or boils in layman’s terms). The fundamental difference between furuncles in otherwise healthy patients and these lesions in HS is that in HS skin the inflammatory response does not subside but instead escalates relatively unabated to form abscesses, sinus tracts, and dermal fibrosis. The results presented herein suggest that defects in cutaneous immune regulation play a major role in disease pathogenesis. Molecular interrogation of clinically and histologically normal appearing nonlesional skin adjacent to HS plaques illuminates pathways that may be predisposed to disease because there is a high potential for this skin to eventually develop HS pathology. We found that indeed this skin was not normal but quantitatively reduced in regulatory myeloid cell subsets, including cDC2s and CD163-expressing macrophages (Figure 2). While CD163-expressing macrophages are known to suppress inflammation in contexts such as cancer, they may potentially encourage fibrosis in HS (58, 59). Reduction within active inflammatory HS lesions followed by a rebound as patients enter end-stage fibrotic disease fits well with this hypothesis. In addition we found that the IL-1 pathway was prominent in HS skin (Figure 1 and Figure 3). IL-1R2 is a major inhibitor of productive IL-1 signaling in tissues (31, 35, 36), and expression of this gene was significantly reduced in nonlesional and lesional HS skin (Figure 2 and Figure 3). Thus, reduced regulatory myeloid cell subsets and defective regulation of IL-1 signaling may predispose to the development of a robust initial inflammatory infiltrate after rupture of follicular cysts in patients with HS. Interestingly, nonlesional HS skin also harbored defects in cellular homeostasis pathways, including apoptosis, metabolism, and DNA damage repair (Figure 2). The relative contribution of hair follicle–associated keratinocytes to this signature is currently unknown; however, if these pathways are fundamentally altered in these cells, it may help explain why this skin is predisposed to undergo follicular occlusion. Indeed, enhanced keratinocyte proliferation and/or reduced cell death in the isthmus/infundibulum region of the hair follicle has been proposed as one of the earliest abnormalities in HS skin (27, 60). In addition, genetic alterations, including gamma-secretase mutations, have been reported in HS patients, which may lead to inappropriate Notch signaling and disruption of normal hair follicle biology (26, 61, 62).

Rupture of follicular cysts in HS patients results in a heterogenous immune cell infiltrate composed initially of predominantly myeloid cells followed by T cells and B cells (63, 64). At the molecular level, almost all inflammatory pathways analyzed were markedly elevated relative to healthy skin (Figure 1). This was quite different from that of psoriasis skin, where the dominant immune pathways were more narrowly focused and significantly reduced in magnitude. Previous reports characterizing immune infiltrates and cytokine expression patterns in HS skin reflect the diversity of inflammation that is present in this disease. These have demonstrated alterations in macrophage and dendritic cell populations, including upregulation of TLR2 expression (65, 66). Plasma cell infiltrates have also been reported in HS lesional skin, which may link to neutrophil pathogenesis through production of neutrophil extracellular trap–specific antibodies (67, 68). In addition, increased IL-17A and IFN-γ production in both CD4^+^ and CD8^+^ T cells have been observed in HS skin (69, 70). Our data are consistent with these findings and further suggest that type 1 T cell responses dominate over IL-17 responses in HS skin. We also show that specific myeloid cell subsets that are normally regulatory in nature adopt a proinflammatory phenotype in inflamed HS lesions, with dysregulation of the IL-1 pathway perhaps lying at the center of this phenomenon. In addition, we found that regulatory T cells are relatively reduced, as percentages of these cells within the CD4^+^ T cell compartment were equivalent to that of normal skin, despite the fact that HS skin is highly inflamed. This is in stark contrast to psoriatic skin and other highly inflamed tissues (49, 50, 52, 53), suggesting that a defective compensatory Treg response may contribute to the ongoing and chronic dysregulated skin inflammation observed in this disease. Interestingly, Tregs localize to hair follicles in skin (71, 72) and have recently been reported to play a major role in suppressing dermal fibrosis (73), a prominent feature of late-stage HS lesions.

Although plasma cells have been observed to infiltrate HS skin (67, 68), the results presented herein reveal memory B cells as a novel component in earlier HS lesions. In addition, we observed that B cells were the major immune cell population attenuated with anti–TNF-α treatment, while IL-1 and T cell responses were relatively unaffected (Figure 7 and Figure 8). Interestingly, treatment of rheumatoid arthritis patients with TNF-α blockade has a significant impact on the B cell compartment (reviewed in ref. 74), suggesting that B cell modulation induced by anti–TNF-α is not limited to patients with HS and is worthy of further investigation because this phenomenon is currently underappreciated and poorly understood. In our study, HS patients who failed to respond to anti–TNF-α treatment contained an enriched B cell signature within HS lesions before initiation of therapy (Figure 9). This may be because skin tissue from these patients was globally more inflamed at the molecular level before treatment, or it may be that disease with a preferentially strong B cell component is less likely to be altered by adalimumab treatment at the currently approved dose. Despite a case report outlining a beneficial effect of B cell depletion in HS (75), HS-like skin disease has also been reported to be induced after B cell–depleting therapy (76). Unfortunately, our cohort was not sufficiently powered to allow us to follow nonresponders with posttreatment samples to determine whether B cell frequencies were successfully reduced within this patient group. Thus, it is currently unclear if B cells significantly contribute to HS pathology or if they accumulate in response to inflammation without playing a major role in driving disease.

TNF-α biology in human skin is complex and currently poorly understood. Multiple immune and nonimmune cell types in skin express mRNA for this cytokine and have the potential to secrete it. In healthy human skin, TNF-α–expressing monocyte/macrophages define a subset of myeloid cells at the single-cell level (Figure 3). Interestingly, this population is reduced in HS skin. In addition, approximately 50% of CD8^+^ and CD4^+^ T cells, and an appreciable number of bona fide Tregs in healthy human skin, produce this cytokine at the protein level after strong T cell receptor stimulation ex vivo (Figure 6 and data not shown). Despite these findings, our analysis revealed that TNF-α was one of the most likely upstream factors driving the gene expression changes observed in HS skin (Figure 1). Furthermore, secretion of this cytokine was significantly increased in CD8^+^ and CD4^+^ T cells in both nonlesional and lesional HS skin (to levels observed in psoriatic skin) (Figure 6). TNF-α can bind to 2 receptors, TNFR1 and TNFR2 (77). While signaling through TNFR1 is thought to mediate the majority of the proinflammatory effects of this cytokine, signaling through TNFR2 has been shown to activate regulatory immune cell populations, including Tregs (78–80). Thus, this cytokine may have opposing effects depending on levels in the tissue microenvironment and the presence of specific cell populations expressing TNFR1 and TNFR2, as well as the relative levels of these receptors on these cells. Consistent with this, numerous patients treated with TNF-α blockade have developed seemingly paradoxical autoimmune or inflammatory diseases, with psoriasis induced by anti–TNF-α being a well-documented side effect of this treatment (81). The effects of TNF-α on both immunostimulatory and immunoregulatory cell subsets may explain why a limited number of HS patients respond to this treatment. Alternatively, it may be that inflammation in HS skin is so heterogeneous and strong in magnitude that blockade of any single factor will have limited efficacy. Although open to interpretation, we feel that our data support the latter because patients with HS with more heightened global inflammatory signatures at baseline were less likely to respond to anti–TNF-α therapy (Figure 9). Further studies are needed to determine if the inflammatory signature identified herein can be used to prospectively stratify patients, in an attempt to improve clinical responses to this treatment.

Given the high number of patients with HS and the morbid nature of this condition, several immunomodulatory treatments currently approved for other indications are being clinically tested in this disease. The data presented here may provide a molecular rationale for the pursuit of some of these while discouraging others, as well as providing a resource for new target discovery. In addition, the ex vivo culture assay we have developed with HS skin may provide a novel platform to functionally test both existing and new immune therapeutics for this disease.

## Methods

### HS study participants.

Fresh 6 mm skin punch biopsies were collected from inflammatory nodules (lesional) and normal appearing skin in the same anatomic region 10 cm away from the lesional tissue (nonlesional). Response to adalimumab was defined using the metric of achievement of HiSCR.

### Psoriatic study participants.

Conventional psoriasis patient samples and site-matched healthy controls were previously reported on in Ahn et al. (28).

### Whole-tissue RNA-sequencing.

Human skin from 16 healthy donors resulting from surgical discards taken from the armpit or groin and HS lesional and nonlesional biopsies were placed in RNA*later* (Invitrogen, Thermo Fisher Scientific) overnight at 4**°**C before banking at –80**°**C before processing by Expression Analysis in 4 batches. Reads were aligned to Ensembl hg19 GRCh37.75 reference genome with kallisto software (v. 0.46.0) using the bootstrap = 40 setting (82). FASTQ files from the 8 conventional psoriasis samples and 9 control samples were realigned by the same method.

### Tissue processing.

Single-cell suspensions of punch biopsies and surgical excisions were obtained by overnight enzymatic digestion with collagenase IV (0.8 mg/mL, Worthington, LS004186) and DNAse (20 μg/mL, MilliporeSigma, DN251G).

### Single-cell RNA-sequencing.

Surgical excisions with pathology consistent with HS and healthy control samples were dermatomed at a 1000 micron depth and were digested overnight, and live, CD3^–^CD19^–^ events were sort purified and loaded onto a 10x Genomics Single Cell 3′ v3 chip. (See Supplemental Methods and Supplemental Figures 19–21.)

### Mass cytometry.

Single-cell suspensions from enzymatically digested skin were banked for CyTOF and frozen, then thawed and stained with antibodies listed in Supplemental Table 1.

### Ex vivo culture.

Ex vivo culture experiments were performed on single-cell suspensions of HS skin by incubating 300,000 cells with suboptimal TCR stimulation (0.1 μg/mL anti-CD3/28, plate bound) for 3 days in media or increasing concentrations of anti-TNF antibody. After 3 days cells were banked and analyzed via CyTOF.

### Flow cytometry.

Samples were stimulated with Cell Stimulation Cocktail (Tonbo Biosciences, catalog TNB-4975) for 4 hours at 37°C, then were stained with antibodies described in Supplemental Table 2. Psoriatic samples were collected contemporaneously with HS samples and have been described in a previous publication (28).

### Data availability.

RNA-Seq data are available at the National Center for Biotechnology Information’s Gene Expression Omnibus database, accession numbers GSE155176 and GSE155850.

### Statistics.

Differential expression within whole-tissue RNA-Seq was performed via the DESeq Wald test. GSVA was analyzed via linear modeling with an empirical Bayes test with the limma package and Benjamini-Hochberg correction. PANTHER pathway analysis was performed via the PANTHER web tool with the Fisher exact test with false discovery rate correction. See Supplemental Table 3. IPA *P* value calculations were performed with default settings (83). xCell scores were analyzed via Mann-Whitney *U* test due to nonparametric distribution. ScRNA-Seq data were analyzed with Wilcoxon’s rank sum test. Comparisons in flow and CyTOF analysis between multiple groups were analyzed via 1-way ANOVA, while 2-group comparisons were made with 2-tailed Student’s *t* test. *P* < 0.05 was considered significant. All figure error bars show mean ± SEM.

### Study approval.

The UCSF Institutional Review Board approved the proposed studies (approval 16-19770). Surgical specimens received were deidentified and certified as Not Human Subjects Research.

## Author contributions

MDR, MML, and HBN conceived and designed the study. HBN, M Paul, HH, EK, USS, and SLH contributed to patient phenotyping and sample acquisition. ML, SC, and M Pauli contributed to data acquisition and analysis. RA and WL contributed data acquisition and analysis of patients with psoriasis. KMS, YB, RD, and JEG contributed to data analysis and interpretation. All authors contributed to the drafting of the manuscript.

## Supplementary Material

Supplemental data

Supplemental Table 1

Supplemental Table 2

Supplemental Table 3

## Version 1 08/25/2020

In-Press Preview

## Version 2 10/02/2020

Electronic publication
