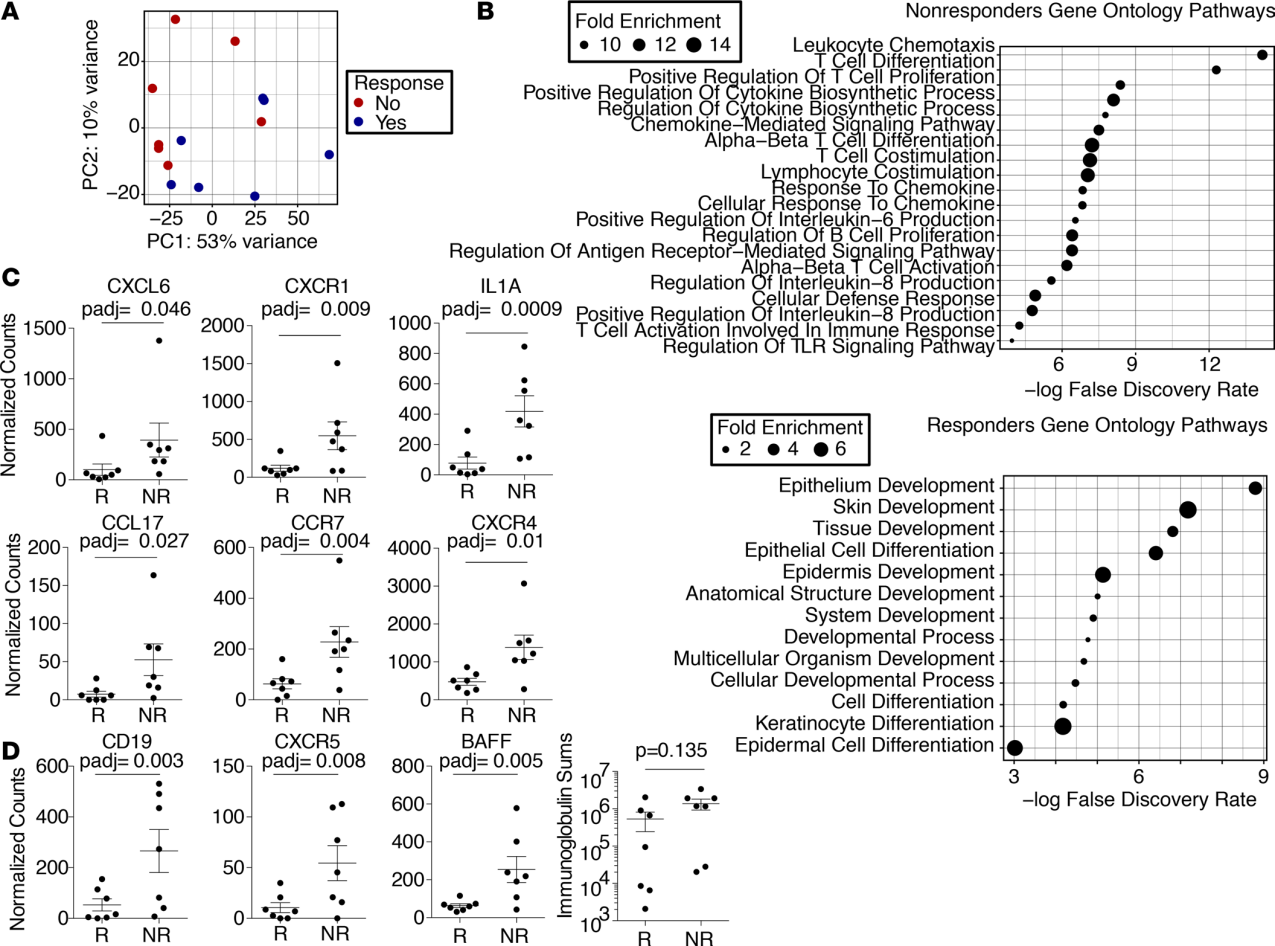# Immunopathogenesis of hidradenitis suppurativa and response to anti–TNF-**α**﻿﻿ therapy

**DOI:** 10.1172/jci.insight.165502

**Published:** 2022-10-24

**Authors:** Margaret M. Lowe, Haley B. Naik, Sean Clancy, Mariela Pauli, Kathleen M. Smith, Yingtao Bi, Robert Dunstan, Johann E. Gudjonsson, Maia Paul, Hobart Harris, Esther Kim, Uk Sok Shin, Richard Ahn, Wilson Liao, Scott L. Hansen, Michael D. Rosenblum

Original citation: *JCI Insight*. 2020;5(19):e139932. https://doi.org/10.1172/jci.insight.139932

Citation for this corrigendum: *JCI Insight*. 2022;7(20):e165502. https://doi.org/10.1172/jci.insight.165502

The authors recently became award of inconsistencies in Figure 9, C and D, and have identified an error in which the data from a nonresponder had been inadvertently plotted in the responder column. The correct figure parts are below. The HTML and PDF versions have been updated online. The authors have stated that all conclusions of the paper remain the same.

The authors regret the errors.

## Figures and Tables

**Figure 9:**