# Supplementary material for: Immunopathogenesis of hidradenitis suppurativa and response to anti–TNF-α therapy
Source: JCI Insight. 2020 Oct 2;5(19):e139932. doi: 10.1172/jci.insight.139932 (PMC7566733; doi:10.1172/jci.insight.139932)
Supplement: Supplemental data [file jciinsight-5-139932-s038.pdf]

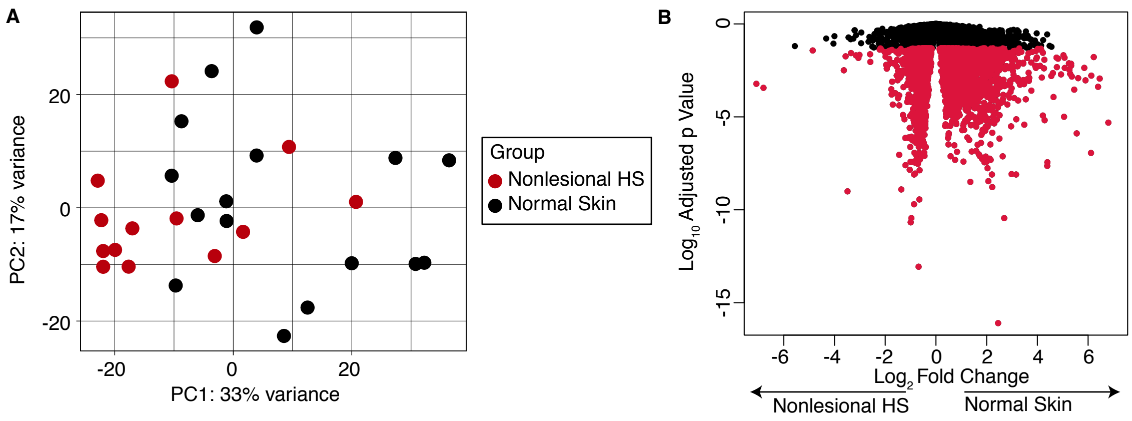

Supplemental Figure 1. **A.** Principal component analysis (PCA) of whole tissue RNA-Sequencing data from nonlesional HS skin and healthy control skin taken prior to initiation of anti-TNF. **B.** Volcano plot showing Log_2 Fold Change and Log_{10} Adjusted P values comparing nonlesional skin to healthy control skin. Transcripts with adjusted p values <0.05 are colored in red.

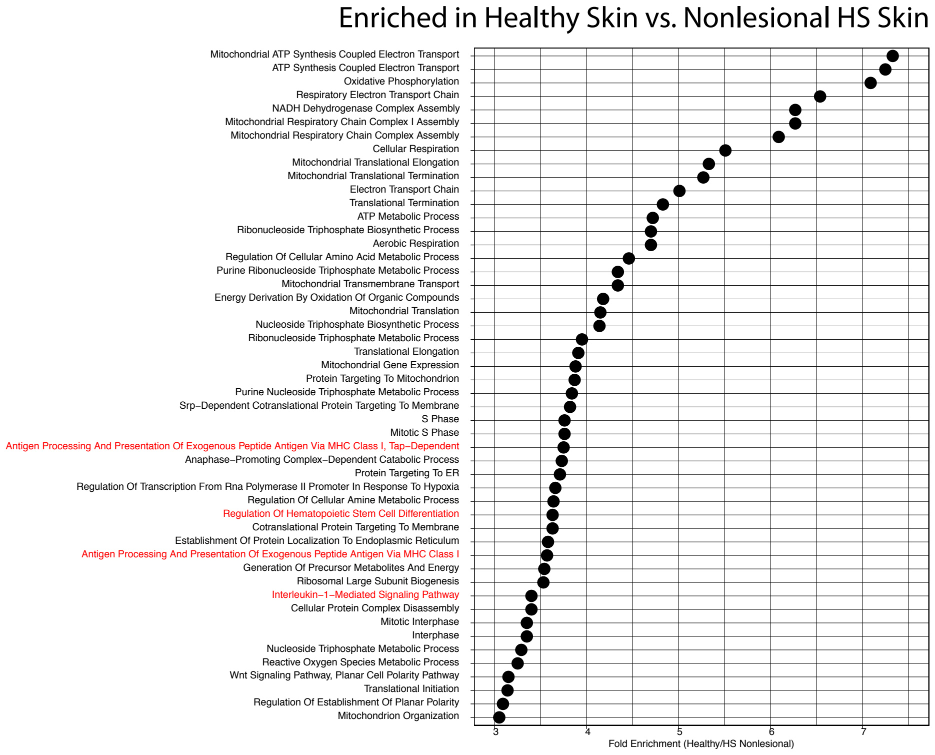

Supplemental Figure 2. The top 50 enriched (FDR<0.05) PANTHER Gene Ontology Pathways identified from genes significantly (adjusted p<0.05) increased in healthy control skin compared to pre-treatment nonlesional HS skin.

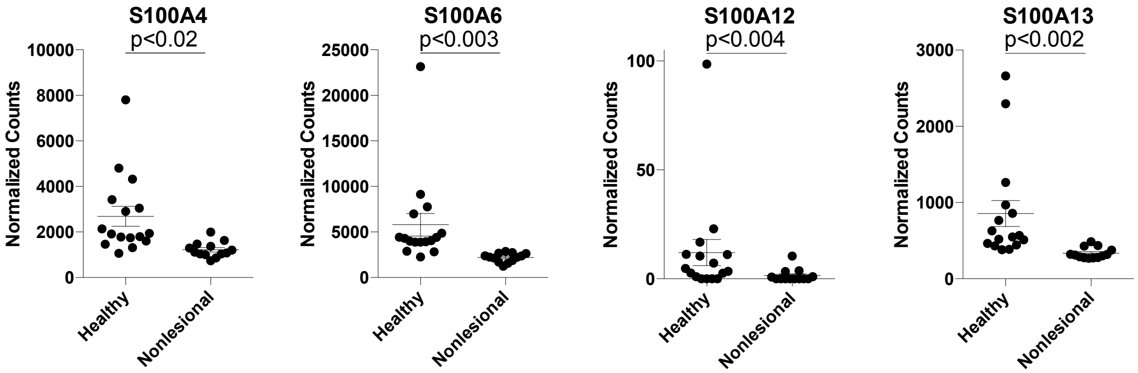

Supplemental Figure 3. Normalized counts for selected antimicrobial peptides in whole tissue RNA-Sequencing of healthy control skin and nonlesional HS skin prior to anti-TNF therapy (Wald test, DESeq).

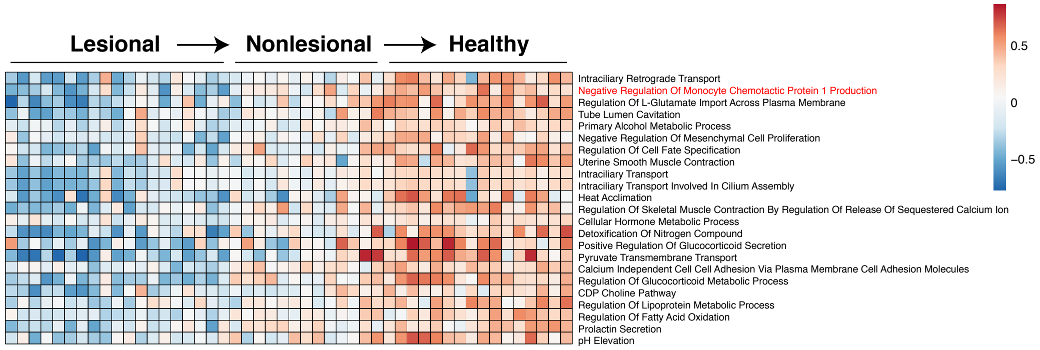

Supplemental Figure 4. GSVA enrichment scores of the union of Gene Ontology pathways significantly decreasing (adjusted p<0.05) in pre-treatment lesional HS skin versus pre-treatment nonlesional HS skin and pre-treatment nonlesional HS skin versus healthy control skin. Each column depicts an individual patient.

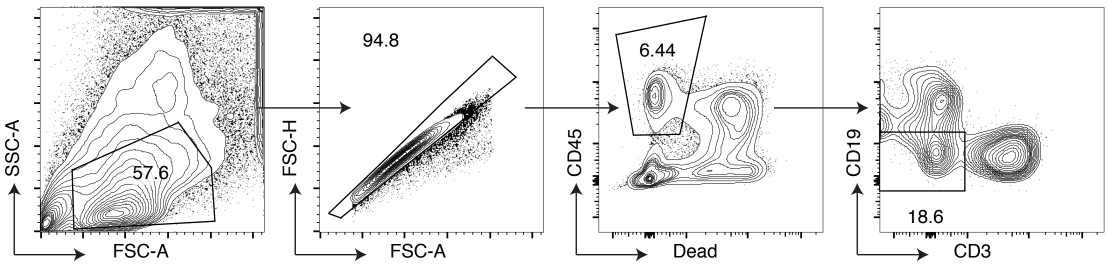

Supplemental Figure 5. Gating strategy for sort-purification of myeloid cells for single-cell RNASeq.

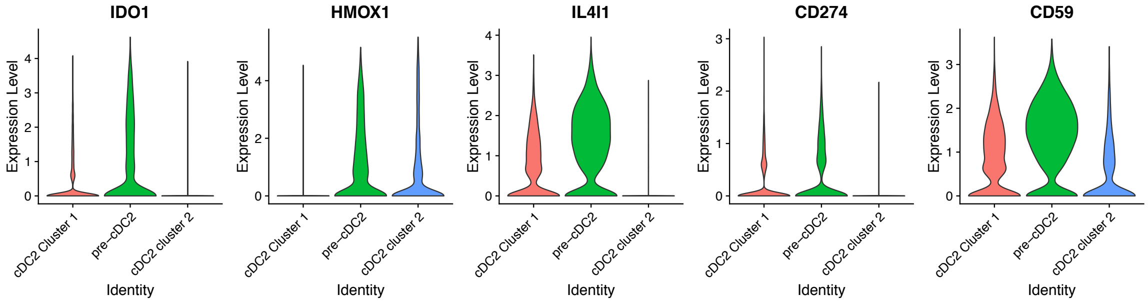

Supplemental Figure 6. Expression of IDO1, HMOX1, IL4I1, CD274 (PD-L1), and CD59 in scRNASeq data of myeloid cells of 2 HS skin samples and 2 healthy skin samples.

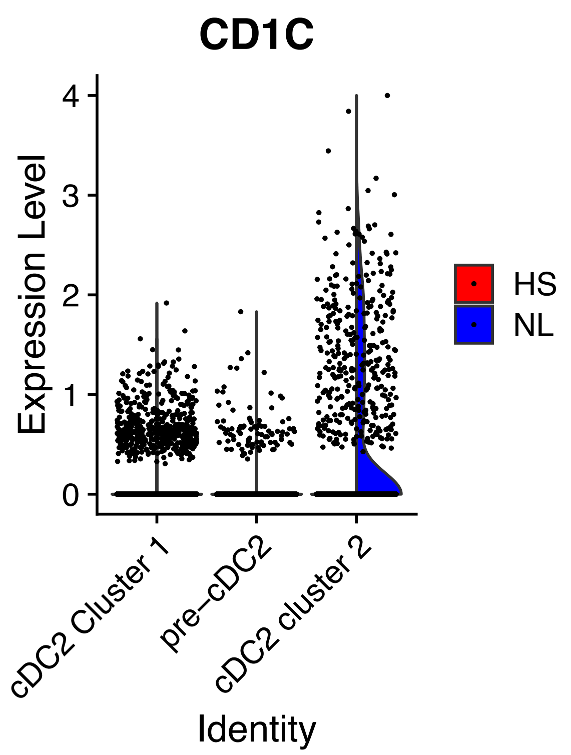

Supplemental Figure 7. CD1C expression on cDC2 and pre-cDC2 clusters in scRNASeq data of myeloid cells of 2 HS skin samples and 2 healthy skin samples.

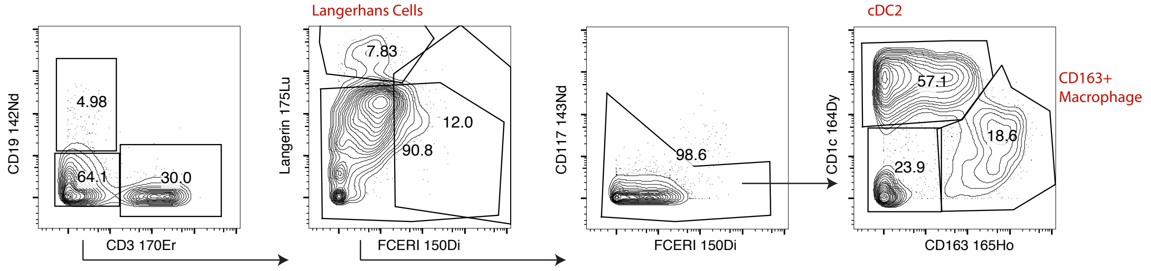

Supplemental Figure 8. Gating strategy for CyTOF analysis of myeloid cells.

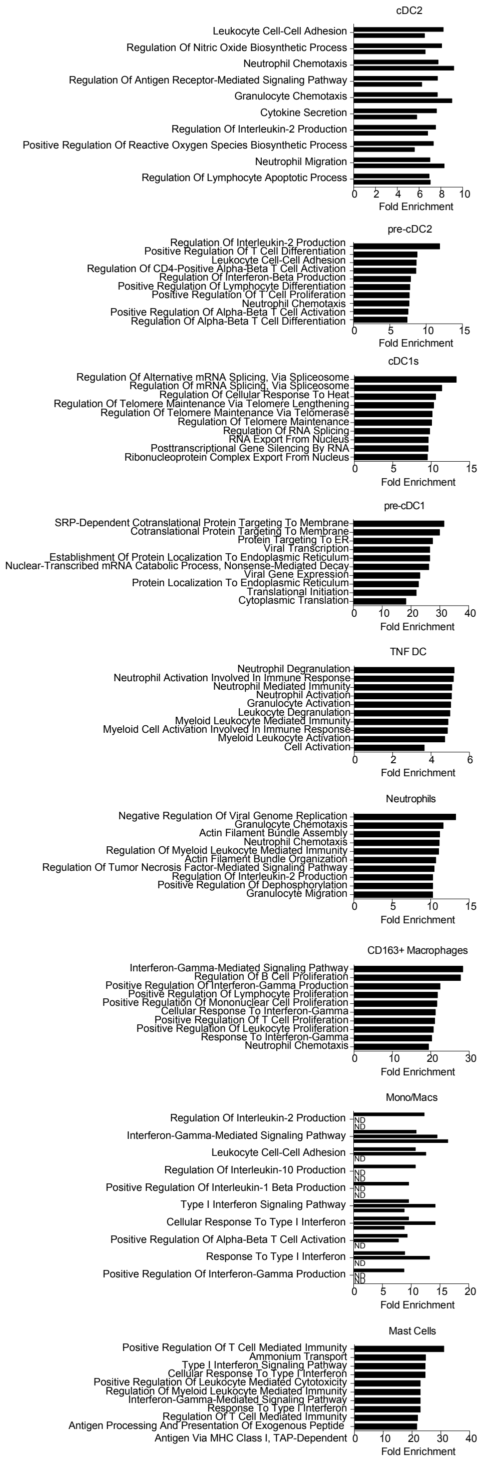

Supplemental Figure 9. Pathway analysis of differentially enriched genes within clusters of single cell RNA-sequencing data comparing healthy skin samples to HS end-stage disease.

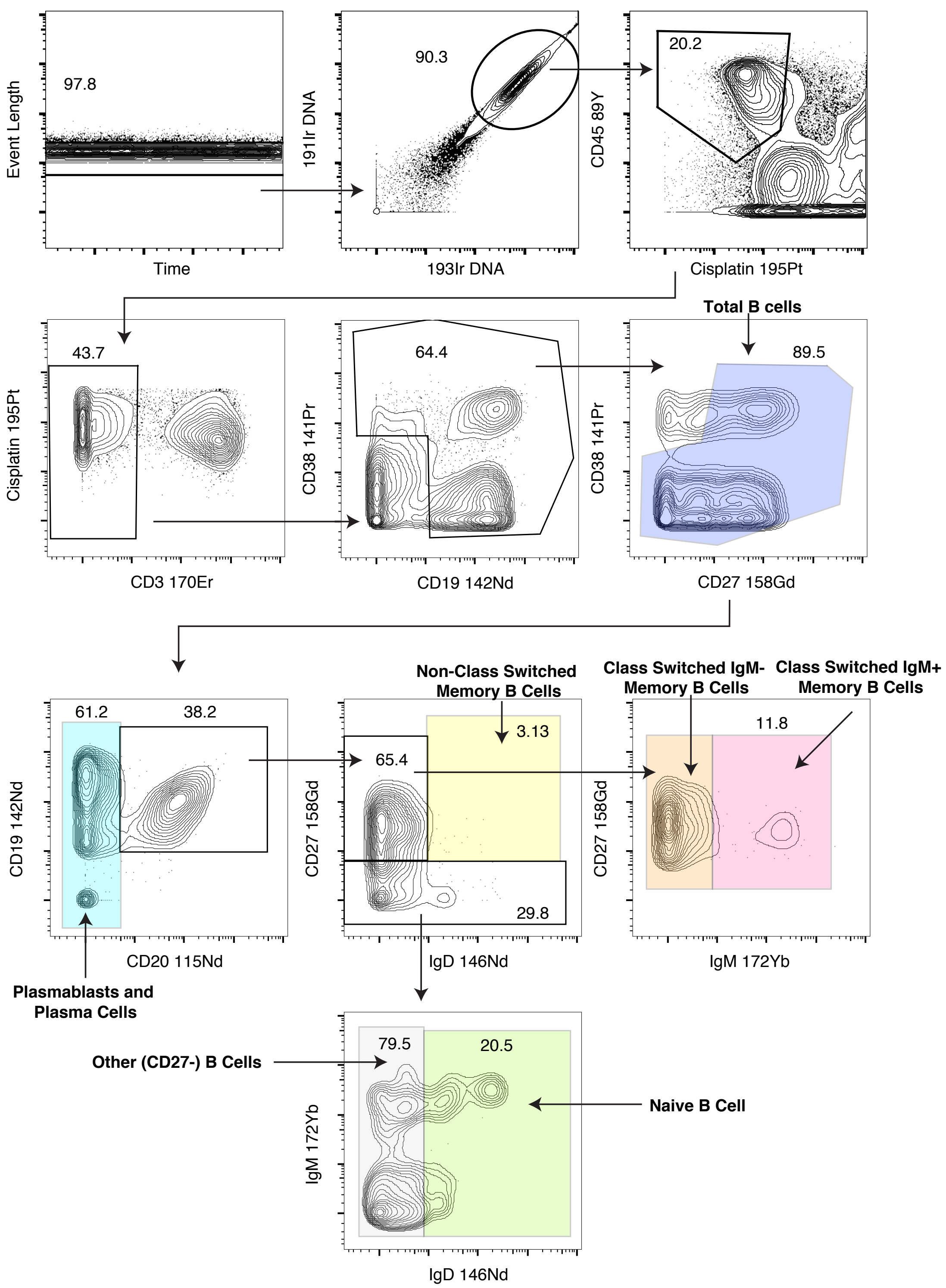

Supplemental Figure 10. Gating strategy for CyTOF analysis of B cells.

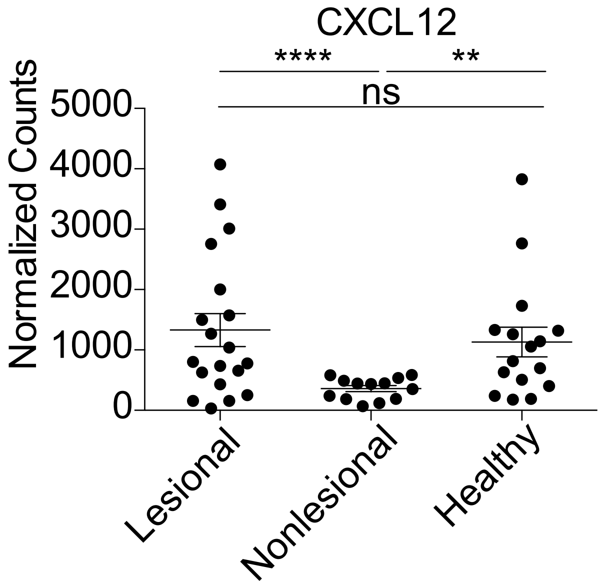

Supplemental Figure 11. Normalized counts for the gene CXCL12 in whole tissue RNA-Sequencing of lesional and nonlesional HS skin prior to anti-TNF therapy compared to healthy control skin (Wald test, DESeq).

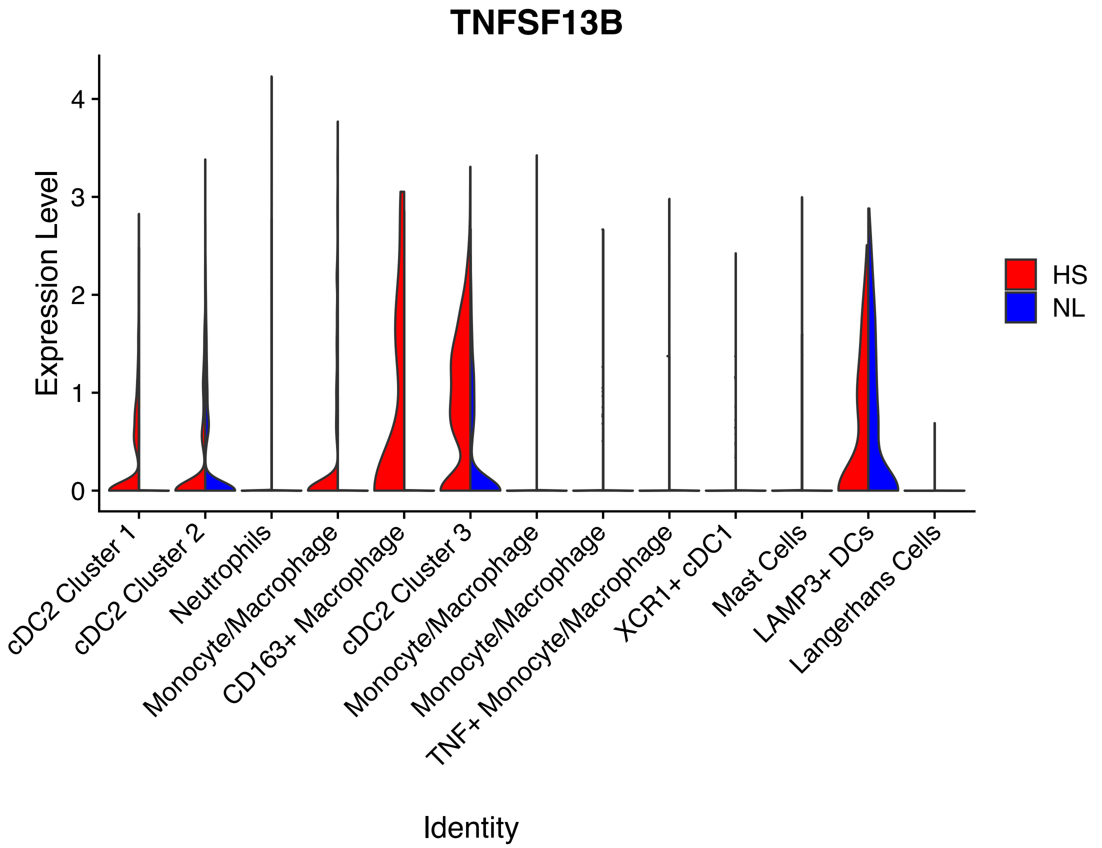

Supplemental Figure 12. Violin plots showing expression of TNFSF13B (corresponding to the protein BAFF) in myeloid clusters of scRNASeq data of two HS skin samples and two normal skin samples.

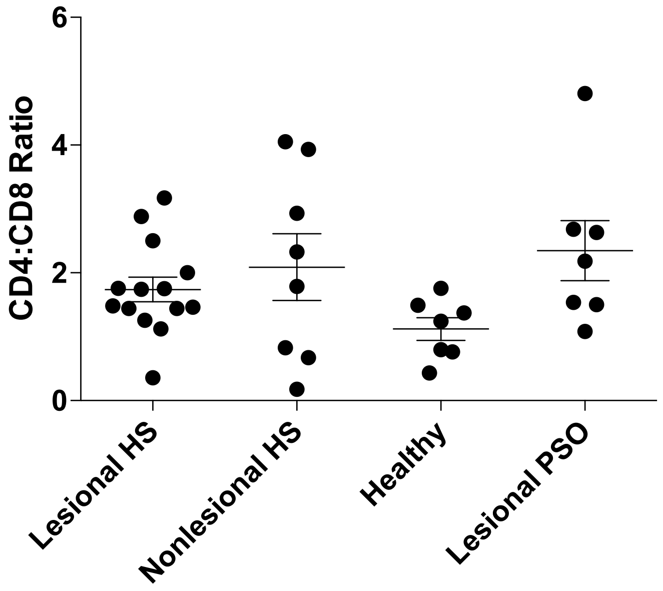

Supplemental Figure 13. Ratios of CD4 T cells to CD8 T cells within lesional HS, nonlesional HS, healthy control, and lesional psoriasis samples. One-way ANOVA, ns.

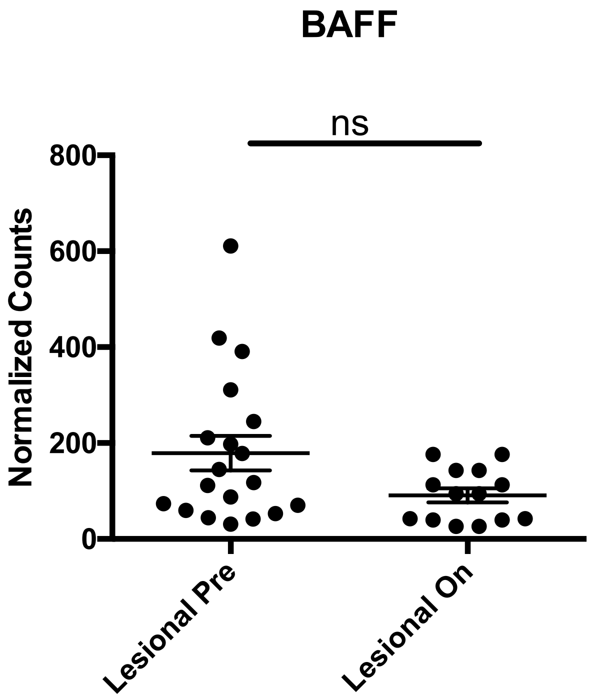

Supplemental Figure 14. Normalized counts of TNFSF13B, correspoding to the protein BAFF, transcript in lesional HS skin prior to anti-TNF therapy versus lesional HS skin from patients on anti-TNF therapy.

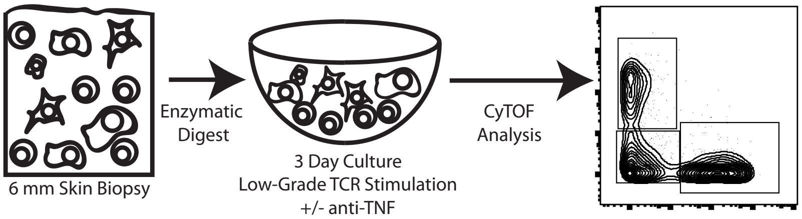

Supplemental Figure 15. Experimental design of HS ex vivo culture assays.

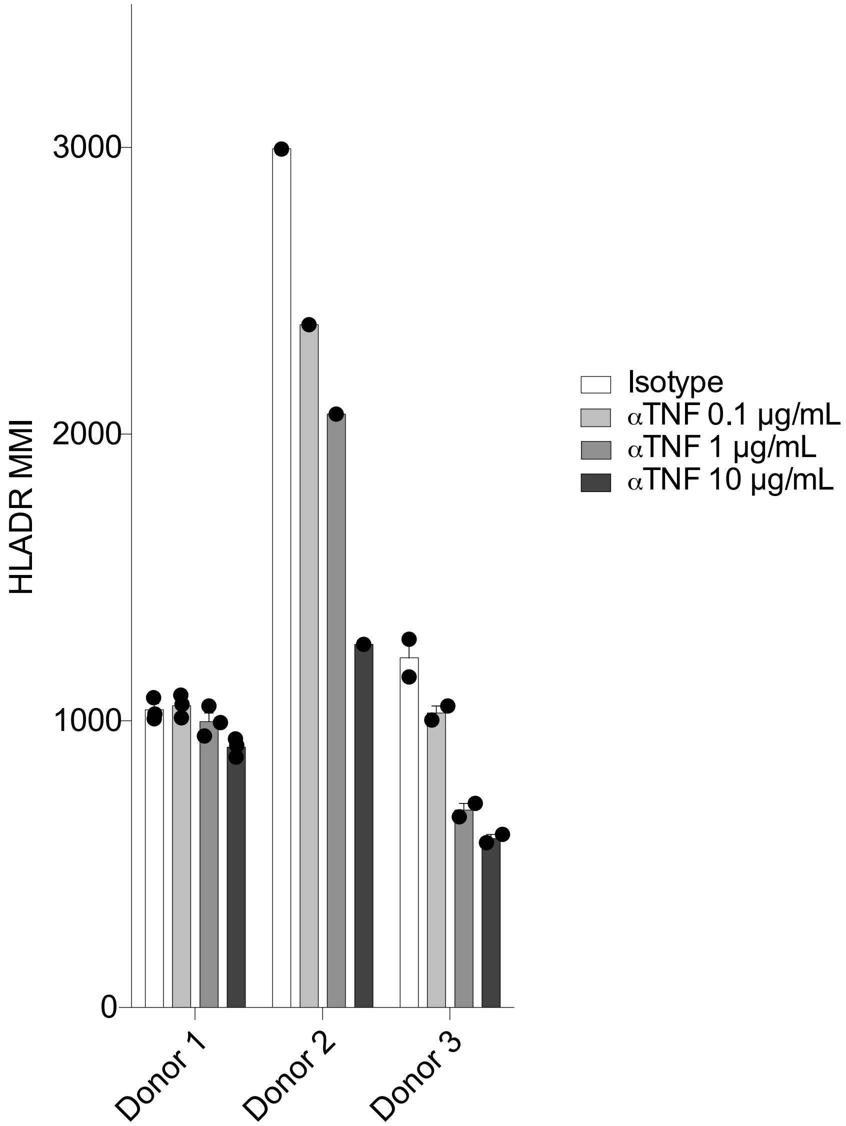

Supplemental Figure 16. HLADR Median Metal Intensity (MMI) on CD19+ B cells from HS skin after three days of culture with isotype control or increasing concentrations of anti-TNF antibody.

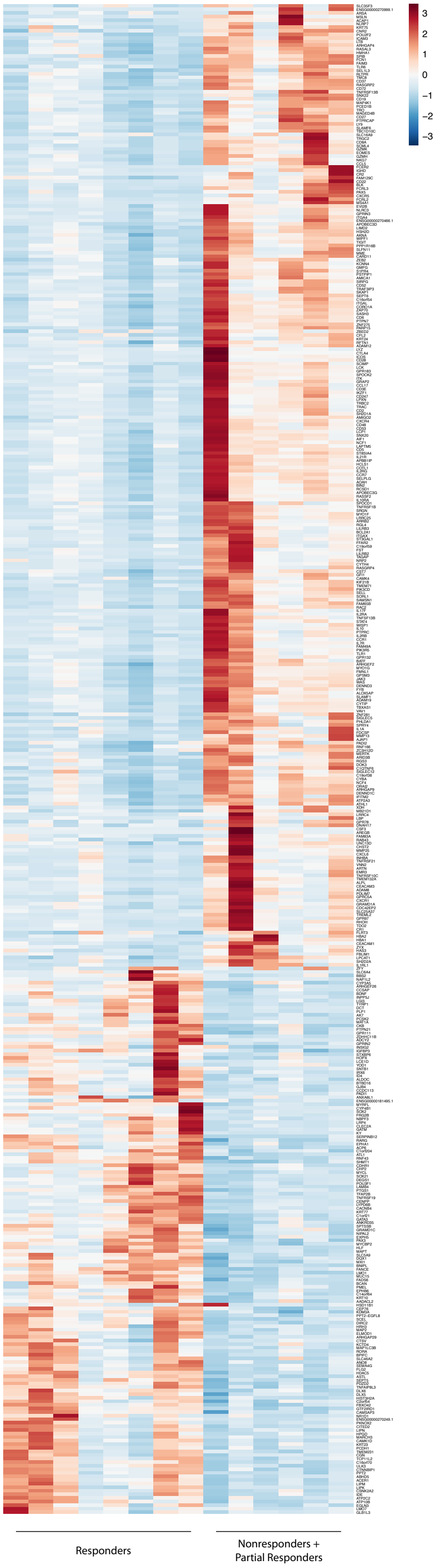

Supplemental Figure 17. Heatmap of genes significantly different between patients responding to anti-TNFα therapy versus those who did not (Wald test, adjusted p<0.05).

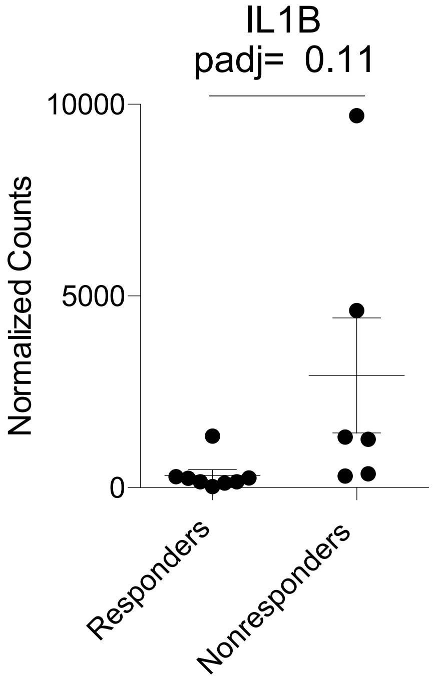

Supplemental Figure 18. Normalized counts for IL1B in whole tissue RNA-Sequencing comparing pre-treatment lesional skin of responders to anti-TNF therapy to non-responders (Wald test).

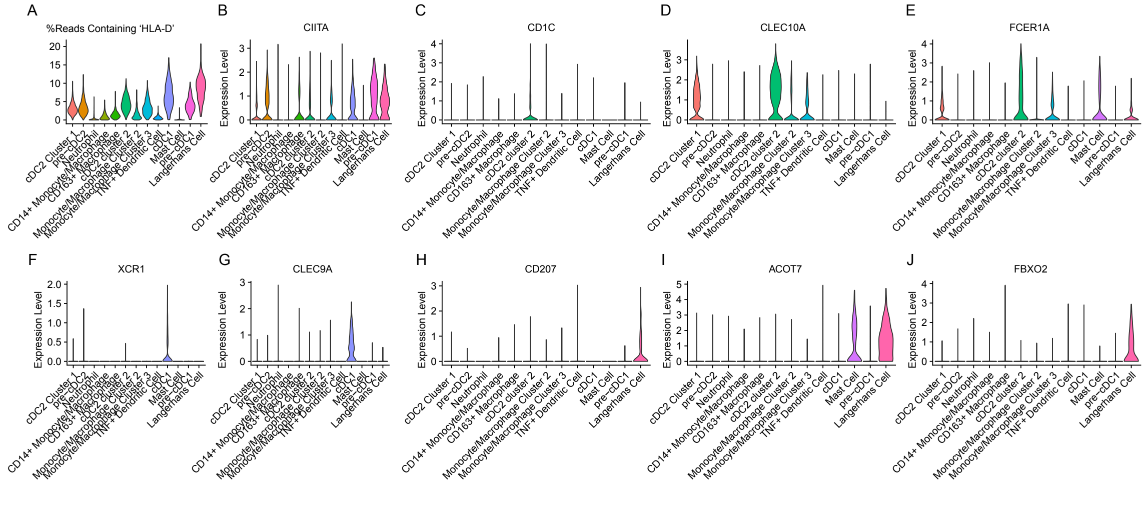

Supplemental Figure 19. Violin plots of combined scRNASeq data of myeloid cells from two HS skin samples and two healthy control skin samples showing (A) Expression of percentage of reads within cells matching the gene pattern 'HLA-D' (B) CIITA, (C)CD1C, (D)CLEC10A, (E)FCER1A, (F)XCR1, (G)CLEC9A, (H)CD207, (I) ACOT7, and (J) FBXO2.

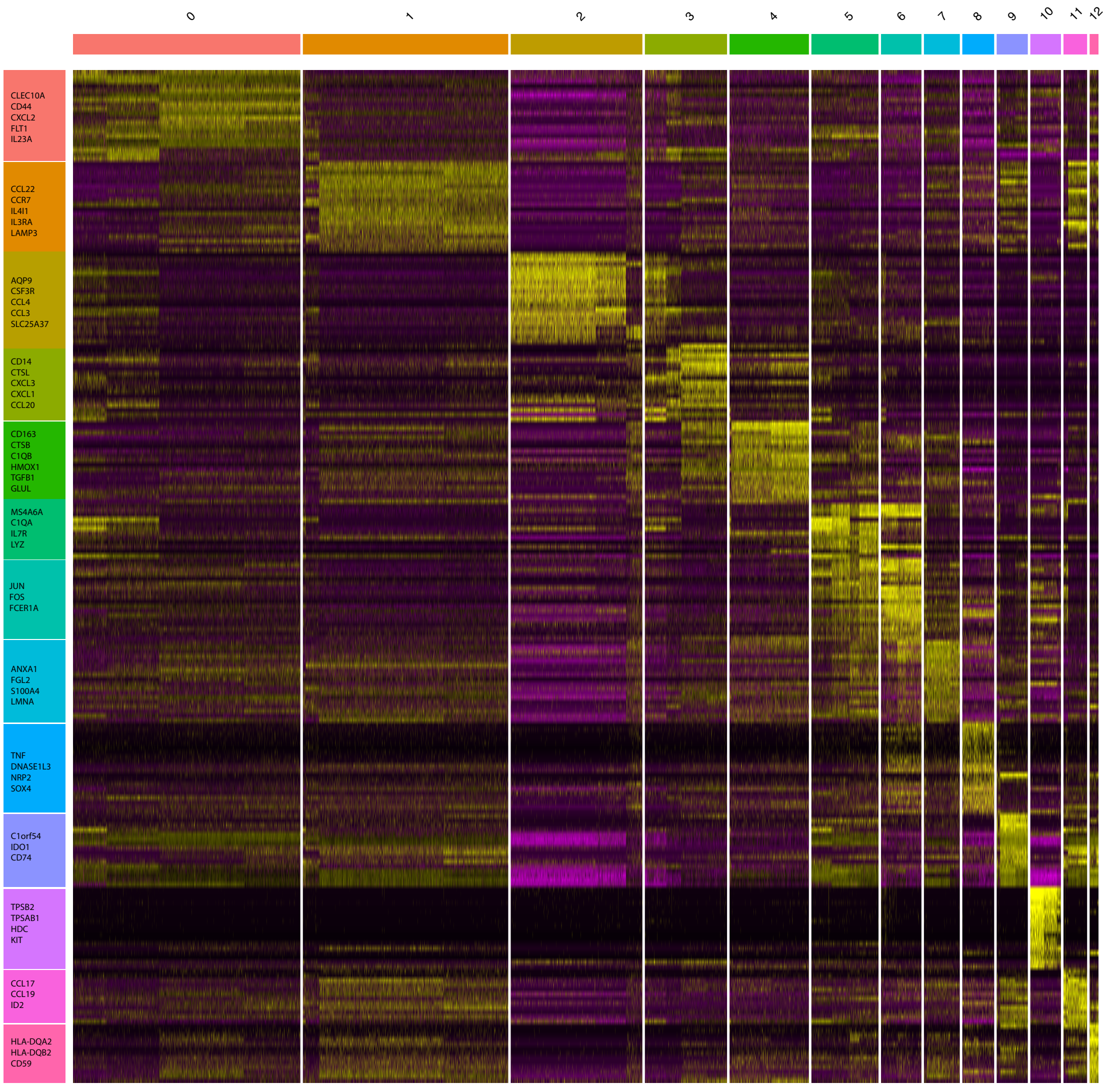

Supplemental Figure 20. Heatmap of top twenty fold increased markers in scRNASeq data of myeloid cells for each cluster, with select genes annotated for each cluster.

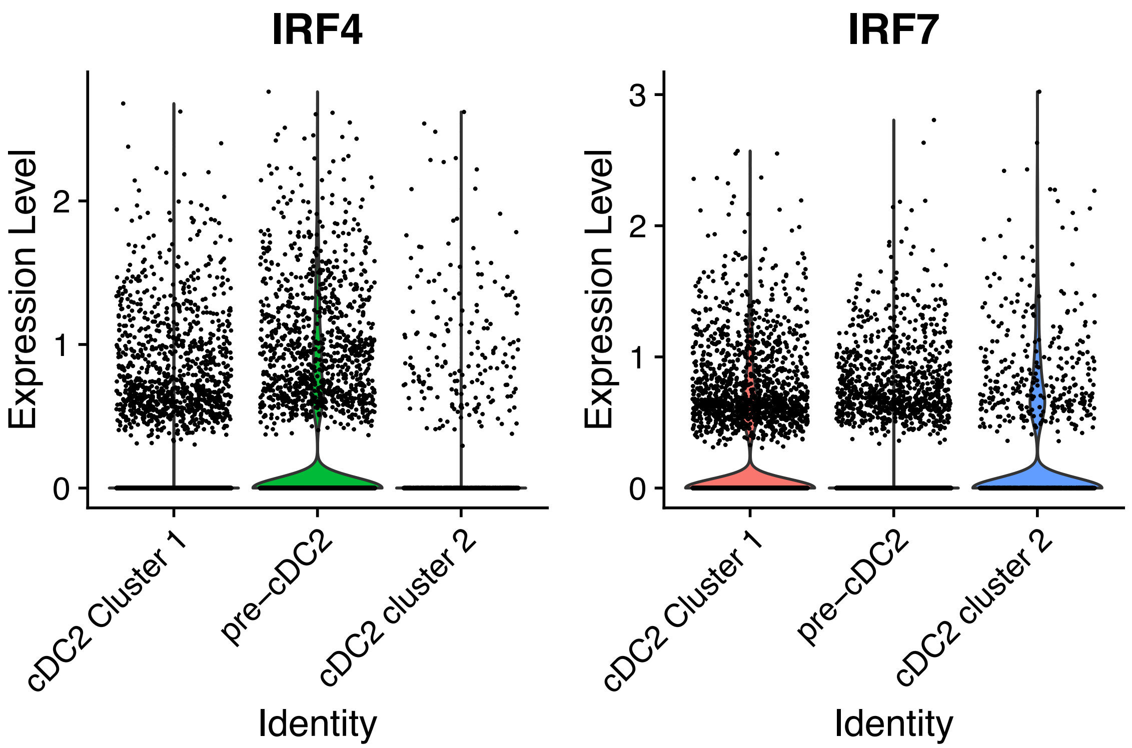

Supplemental Figure 21. Violin plots of IRF4 (left) and IRF7 (right) expression on cDC2 and pre-cDC2 clusters in scRNASeq data of myeloid cells of 2 HS skin samples and 2 healthy skin samples.
